# Multisite Phosphorylation Modulates the T Cell Receptor *ζ*-Chain Potency but not the Switchlike Response

**DOI:** 10.1016/j.bpj.2016.03.024

**Published:** 2016-04-26

**Authors:** Himadri Mukhopadhyay, Ben de Wet, Lara Clemens, Philip K. Maini, Jun Allard, P. Anton van der Merwe, Omer Dushek

**Affiliations:** 1 Sir William Dunn School of Pathology, Mathematical Institute, University of Oxford, Oxfordshire, United Kingdom; 2 Wolfson Centre for Mathematical Biology, Mathematical Institute, University of Oxford, Oxfordshire, United Kingdom; 3 Department of Mathematics, University of California-Irvine, Irvine, California

## Abstract

Multisite phosphorylation is ubiquitous in cellular signaling and is thought to provide signaling proteins with additional regulatory mechanisms. Indeed, mathematical models have revealed a large number of mechanisms by which multisite phosphorylation can produce switchlike responses. The T cell antigen receptor (TCR) is a multisubunit receptor on the surface of T cells that is a prototypical multisite substrate as it contains 20 sites that are distributed on 10 conserved immunoreceptor tyrosine-based activation motifs (ITAMs). The TCR *ζ*-chain is a homodimer subunit that contains six ITAMs (12 sites) and exhibits a number of properties that are predicted to be sufficient for a switchlike response. We have used cellular reconstitution to systematically study multisite phosphorylation of the TCR *ζ*-chain. We find that multisite phosphorylation proceeds by a nonsequential random mechanism, and find no evidence that multiple ITAMs modulate a switchlike response but do find that they alter receptor potency and maximum phosphorylation. Modulation of receptor potency can be explained by a reduction in molecular entropy of the disordered *ζ*-chain upon phosphorylation. We further find that the tyrosine kinase ZAP-70 increases receptor potency but does not modulate the switchlike response. In contrast to other multisite proteins, where phosphorylations act in strong concert to modulate protein function, we suggest that the multiple ITAMs on the TCR function mainly to amplify subsequent signaling.

## Introduction

Protein phosphorylation is a ubiquitous mechanism of signal transduction that is regulated by the opposing actions of kinases and phosphatases (1, 2). Failure to regulate phosphorylation can lead to a number of abnormal cellular phenotypes, such as constitutive cell growth, which is the case in many cancers (1, 3). The majority of eukarotyic proteins are phosphorylated on more than one site (4, 5), raising the possibility that multisite phosphorylation may confer an additional regulatory mechanism (6, 7).

A well-known regulatory mechanism is a switchlike response, whereby the phosphorylation state of a protein can be highly sensitive to the concentrations of its modifying kinase and phosphatase. A large body of work has focused on understanding whether multiple phosphorylation sites on a single substrate can confer switchlike responses. Mathematical models have shown that switchlike responses can be produced by a number of mechanisms that rely on multiple phosphorylation sites, such as substrate sequestration (8), nonessential sites (9), local concentrations (10), independent binding sites (11), membrane-anchoring (diffusion-limited reactions) (12), entropic mechanisms (13), binding of effector molecules (14), cooperativity between sites, or site-specific enzymatic rates (15), and other mechanisms reviewed elsewhere (2, 6, 7). The key difference between the models lies in the assumptions about how the sites are modified. For example, a switchlike response can be produced when the modifying enzymes phosphorylate the substrate in a precise sequence but not when modification is random (Fig. S1 in the Supporting Material). Despite extensive mathematical studies, there are few experimental studies aimed at a systematic investigation of multisite phosphorylation.

The T cell antigen receptor (TCR) on the surface of T cells is a prototypical multisite substrate (16). T cells rely on the TCR to recognize antigens, in the form of peptides bound to major histocompatibility complexes, on the surfaces of antigen-presenting cells. Ligation of the TCR by peptides bound to major histocompatibility complexes can trigger a large signal transduction cascade in T cells that may lead to a number of functional responses, such as proliferation, differentiation, and the secretion of effector cytokines (17, 18). The T cell response is of crucial importance in the elimination of infections and pathologies, and the ability to harness this response is a central objective in immune-based therapies (19).

The TCR is a multisubunit receptor that contains two ligand binding chains that associate with six signaling chains that collectively contain 20 phosphorylation sites. These sites are distributed on 10 immunotyrosine-activated-based motifs (ITAMs) with consensus sequence YXX[L/I]X_6–9_YXX[L/I], where *X* is any amino acid (20). While four of the signaling chains each contain one ITAM, the T cell receptor *ζ*-chains, which form a homodimer by a disulfide bond in the transmembrane domain, each contain three ITAMs (or six ITAMs across the dimer). These sites are phosphorylated by the membrane-anchored tyrosine kinase Lck and Fyn and dephosphorylated by the transmembrane phosphatases CD148 and CD45. When both tyrosines in an ITAM are phosphorylated they generate docking sites for the tandem SH2 domains of the cytosolic tyrosine kinase ZAP-70. Bound ZAP-70 can phosphorylate tyrosines on other substrates that initiate the signal transduction that may lead to T cell activation (17).

Given that both kinases and phosphatases are constitutively active in resting T cells (16, 21), it is thought that the phosphorylation state of the TCR is tightly regulated. This is underlined by the observation that even a single TCR ligand is sufficient to activate T cells (22).

A number of the multisite phosphorylation mechanisms that produce switchlike responses discussed above may be applicable to the T cell receptor *ζ*-chain. The *ζ*-chain has been shown to be sequentially phosphorylated (23, 24), which is sufficient to produce switchlike responses (Fig. S1). Moreover, it contains nonessential sites because even a single phosphorylated ITAM is sufficient to recruit and activate ZAP-70, it has the potential to be sequestered in the membrane when fully dephosphorylated (25), and components of the signaling module are membrane-anchored. We have also used a mathematical model to show that differential binding affinities of ZAP-70 to each of the *ζ*-chain ITAMs combined with sequential phosphorylation can produce switchlike responses (14).

In this work, we have investigated how the phosphorylation state of the *ζ*-chain is regulated by changes to the kinase/phosphatase ratio as the number of phosphorylation sites is modified. To do this, we performed cellular reconstitution of 21 distinct signaling modules that include different combinations of wild-type or mutant forms of the TCR *ζ*-chain, Lck, CD148, and ZAP-70. By reconstituting *ζ*-chains with all combinations of ITAM mutants, we found no evidence for sequential phosphorylation, and surprisingly, we found that phosphorylation sites did not modulate the switchlike response but did modulate receptor potency and maximum phosphorylation. We show that a phosphorylation-dependent enhancement in the binding rate of the enzymes can explain these observations and that a molecular mechanism that is consistent with such an enhancement is a disorder-to-order transition of the *ζ*-chain upon phosphorylation.

## Materials and Methods

### Plasmids

All sequences used in this study were of murine origin unless otherwise stated. TCR *ζ*-chain variants were generated as previously described in Choudhuri et al. (26). Briefly, the transmembrane and cytoplasmic domains of mouse TCR *ζ*-chain were cloned in frame with the extracellular domains of rat CD2 in the mammalian expression vector pcDNA3.1(+). For each ITAM mutant, both ITAM tyrosines were mutated to phenylalanine. Lck was cloned into the mammalian expression vector pEF3 as a constitutively active Y505F-variant (27). An SH2-binding mutant, Lck(R154K), was generated by QuikChange PCR (Agilent Technologies, Santa Clara, CA). Ectodomain-truncated CD148, encoding the last two C-terminal fibronectin type III, transmembrane, and catalytic domains, as well as an extracellular FLAG-tag, was generated as previously described in Cordoba et al. (28), and cloned in the expression vector pcDNA3.1(+). ZAP-70 was cloned into a pcDNA3.1(+) vector, and the low activity variant (ZAP-70^∗^) containing the SH2-binding mutations, R41K and R190K, as well as the catalytically dead mutation, K368A, was generated by QuikChange PCR (Agilent Technologies).

### Cell culture and transfection

HEK 293T cells were cultured at 37°C and in a 10% CO_2_ in DMEM containing 10% fetal bovine serum. A day before transfection, cells were seeded to reach 70% confluence, and a single 175 cm^2^ flask was transiently transfected for each experimental condition. For experiments including only Lck, CD148, and TCR *ζ*-chain variants, 8 *μ*g of each plasmid (24 *μ*g total) was complexed with 24 *μ*g of branched-chain PEI (polyethylenimine, average *M*_*w*_ 25,000; Sigma-Aldrich, St. Louis, MO) in 0.9 mL serum-free medium for 10 min, before dropwise addition to cells in serum-free medium. An additional 25 *μ*g of ZAP-70 DNA and 25 *μ*g of PEI was included in transfection mixtures in experiments containing ZAP70. Cells were incubated at 37°C for 4 h before transfection medium was replaced with complete medium supplemented with fetal bovine serum. Cells were harvested after 24 h by gentle shaking, centrifuged, and resuspended in fresh serum-free medium at an appropriate density for assay.

### Pervanadate treatment of transfectants

Basal kinase/phosphatase balance was disrupted by inhibition of phosphatase activity with a serial dilution of pervanadate. Pervanadate was generated immediately before the assay by incubating 10 mM sodium orthovanadate with 0.7% H_2_O_2_ at room temperature for 10 min. H_2_O_2_ was inactivated by the addition of 20 *μ*g/mL catalase at room temperature for 10 min. A 24-step, 1.25-fold serial dilution of pervanadate was made up in serum-free medium starting at 1 mM. Harvested transfectants were plated at 300,000 cells per well in 96-well assay plates in a volume of 60 *μ*L/well and treated with 60 *μ*L/well pervanadate serial dilutions for 30 min at 37°C in a tissue culture incubator. Reactions were terminated and cells were lysed by adding 120 *μ*L/well lysis buffer containing 2% Nonidet P-40 substitute (Roche, Indianapolis, IN), Mammalian Protease Inhibitor Cocktail (Sigma-Aldrich), and 250 *μ*M sodium orthovanadate in TBS (20 mM Tris, pH 7.5, 150 mM NaCl). Cellular debris was removed by centrifugation and lysates were transferred to wells of previously prepared immunosorbent capture plates.

### Immunosorbent assay of TCR *ζ*-chain phosphorylation

TCR *ζ*-chain phosphorylation was quantified in immunosorbent assay by capturing chimeric TCR *ζ*-chain with anti-rat CD2 antibody and detecting phosphorylation with anti-phosphotyrosine antibody. A day before assay, MaxiSorp flat-bottom 96-well plates (Nalge Nunc, Rochester, NY) were coated overnight at 4°C with 100 *μ*L/well anti-rat CD2 antibody (AbD Serotec) at 0.7 *μ*g/mL in carbonate-bicarbonate buffer, pH 9.6. On the day of the assay, capture antibody was decanted and wells were blocked with 200 *μ*L 1% bovine serum albumin in TBS for 1 h at room temperature. Plates were washed three times with TBS-T buffer (TBS containing 0.05% TWEEN-20). Cleared saturating amounts of lysate was transferred to plates and TCR *ζ*-chain captured for 2 h at 4°C. Lysate was decanted and plates were washed 6 times with TBS-T. Phosphorylation was probed by addition of 100 *μ*L/well of biotinylated anti-phosphotyrosine antibody (clone pY20; BioLegend, San Diego, CA) at 1 *μ*g/mL, and plates were incubated for 1 h at room temperature. Plates were subsequently washed 3 times with TBS-T. Biotinylated antibody was probed with 100 *μ*L/well of 0.1 *μ*g/mL IRDye 800CW-streptavidin (LI-COR Biosciences, Lincoln, NE) for 1 h at room temperature. Plates were washed three times with TBS-T, dried, and fluorescence-detected by scanning plates in an Odyssey Sa Infrared Imaging System (LI-COR).

### Flow cytometry

Transfectants were blocked by incubating 1 × 10^6^ cells for 15 min on ice in 100 *μ*L 1% PBS-A (albumin in phosphate-buffered saline). Surface antigens were stained by incubating for 1 h on ice with 1 *μ*g/ml FITC-conjugated anti-FLAG antibody (Sigma-Aldrich) and 1 *μ*g/mL phycoerythrin-conjugated anti-rat CD2 (AbD Serotec, Kidlington, Oxfordshire, UK) in 100 *μ*L PBS-A. Cells were washed three times in PBS and fixed with 4% paraformaldehyde in PBS for 15 min at room temperature. Cells were washed three times in PBS and permeabilized in 0.1% saponin (Sigma-Aldrich) in PBS-A. Intracellular antigens were stained by incubating for 1 h at room temperature with 1 *μ*g/mL Alexa Fluor 647-conjugated anti-mouse Lck (BD Biosciences, San Jose, CA) and 1 *μ*g/mL Pacific Blue-conjugated anti-mouse ZAP-70 (Thermo Fisher Scientific, Guilford, CT) in 100 *μ*L PBS-A. Cells were washed three times with PBS and analyzed using a FACScan flow cytometer (Becton Dickinson, Franklin Lakes, NJ).

### Curve fitting, normalization, and statistical analyses

The steady-state phosphorylation profiles were fit to a logarithmic Hill function in Prism (GraphPad Software, La Jolla, CA), that produces estimates of *E*_min_ and *E*_max_, which correspond to the minimum and maximum values, respectively, log_10_(*EC*_50_), which is the logarithmic value of the kinase/phosphatase that yields half of the maximal response (otherwise known as potency), and *n*, which is the Hill number that determines the degree of the switchlike response (also known as the sensitivity).

We used an index module (wild-type *ζ* 123) as our standard curve so that experimental data can be averaged across different days. This normalization was performed by (1) dividing the response for each module by the value of *E*_max_ for the index module and (2) by subtracting the pervanadate concentration series on a log-scale (*x* axis) by the value of *EC*_50_ for the index module. Fitted Hill numbers were normalized to the value of the index module.

All statistical analyses were carried out on normalized averaged quantities using a *t*-test in Prism (GraphPad Software). Given that the Hill number and log_10_(*EC*_50_) of the index module was 1 and 0, respectively, we used a *t*-test to determine whether the corresponding values for the group of single ITAM mutants (*ζ*X23, *ζ*1X3, and *ζ*12X) or the double ITAM mutants (*ζ*1XX, *ζ*X2X, and *ζ*XX3) differed from these values. An unpaired *t*-test was used to compare between the group of single and double ITAM mutants.

## Results

### Cellular reconstitution of multisite phosphorylation of the T cell receptor *ζ*-chain

**Figure 1:**
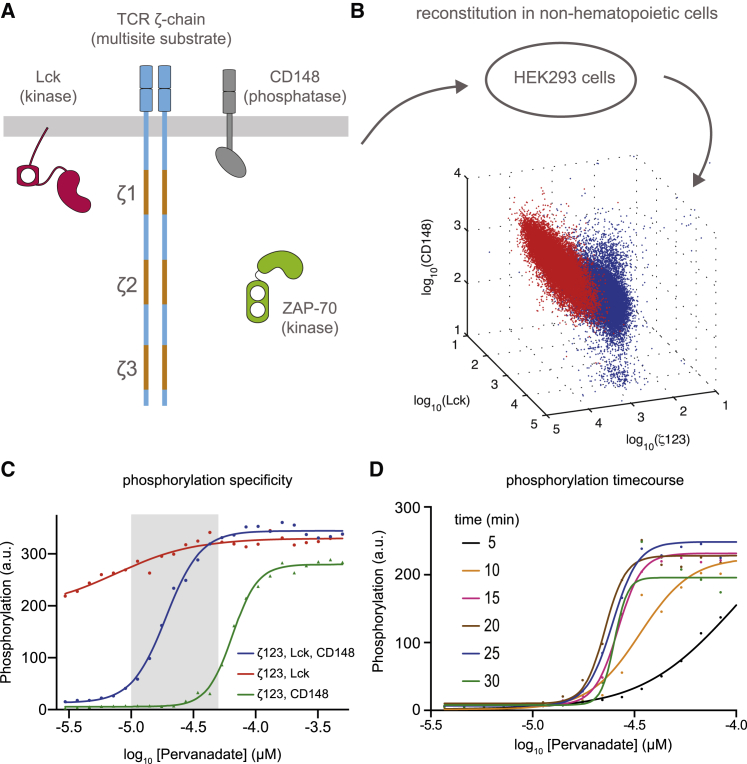
Cellular reconstitution of multisite phosphorylation of the T cell receptor *ζ*-chain. (*A*) Schematic of reconstituted signaling proteins. The substrate is a CD2-TCR*ζ*-chain chimera that contains six phosphorylation sites distributed on three ITAMs (*orange*) that dimerizes as a result of a disulfide bond in the *ζ*-chain transmembrane domain (i.e., the substrate is a receptor dimer that contains 12 phosphorylation sites). The substrate is phosphorylated by the membrane-anchored kinase Lck and dephosphorylated by the transmembrane phosphatase CD148. The cytosolic kinase ZAP-70 can bind to phosphorylated ITAMs by SH2 domains. (*B*) Combinations of these components were transfected into the nonhematopoietic HEK293 cell line and molecular expression was detected with flow cytometry 24 h posttransfection. A *k*-means clustering algorithm classified the cells as either positive (*red*, 25%) or negative (*blue*, 75%) for transfected components (see Fig. S2 for two-dimensional projections). (*C*) Cells transfected with the indicated components were incubated with increasing concentrations of the tyrosine phosphatase inhibitor pervanadate for 30 min (*x* axis) before total phosphorylation of the *ζ*-chain was determined (*shaded rectangle* highlights the specificity range). (*D*) Phosphorylation time course for reconstitution of Lck, CD148, and *ζ*-chain indicates that steady-state phosphorylation is achieved by ∼15–20 min. See Materials and Methods for experimental details. To see this figure in color, go online.

To study the effects of multiple phosphorylation sites on the T cell receptor *ζ*-chain, we reconstituted four components in various combinations (Fig. 1 *A*). The wild-type substrate was a chimera consisting of the *ζ*-chain transmembrane and cytosolic domains but with the extracellular domain of rat CD2 (previously used as a simplified receptor (26)). The wild-type kinase was Lck with a point mutation to a negative regulatory tyrosine (Y505F) that renders the kinase constitutively active. The wild-type phosphatase was CD148 with a truncated extracellular domain, which was previously shown to have higher expression (28). Lastly, we have used wild-type ZAP-70 in a number of experiments.

Combinations of the wild-type components or mutant variants were transfected into HEK293 cells (Fig. 1 *B*), which comprise a nonhematopoietic human cell line that is not expected to express any of the endogenous T cell receptor proximal signaling proteins (29). We found that expression peaked 24 h posttransfection (not shown), and therefore all experiments were performed at this time point. Using a *k*-means clustering algorithm, we found that cells were readily classified into two distinct populations that were either positive for all three components or were negative for all three components (Fig. 1 *B*). Moreover, component expression in the positive population was correlated (Fig. S2), which has been previously shown to be the case in transfections of HEK293 cells (30).

To determine the total phosphorylation profile of the *ζ*-chain, the cells were treated with a titration of the tyrosine phosphatase inhibitor pervanadate for 30 min before the *ζ*-chain was captured using an anti-CD2 antibody and phosphorylation detected using an anti-phosphotyrosine antibody (Fig. 1 *C*). We found limited phosphorylation without pervanadate treatment and moreover, treatment with the Lck inhibitor PP2 did not lead to detectable reduction in phosphorylation (not shown), suggesting that CD148 dominates over Lck basally. Given that HEK293 cells are expected to have endogenous tyrosine kinases and phosphatases, we determined their contribution by transfecting in the substrate with only Lck or only CD148. Although we observe dephosphorylation in the absence of exogenous CD148 or phosphorylation in the absence of exogenous Lck, a large range exists where phosphorylation is regulated primarily by exogenous Lck and CD148 (*shaded region*, Fig. 1 *C*). Moreover, a timecourse revealed that by ∼15–20 min, the system has come to steady state (Fig. 1 *D*).

### Multisite phosphorylation of the T cell receptor *ζ*-chain modulates potency but not the switchlike response

**Figure 2:**
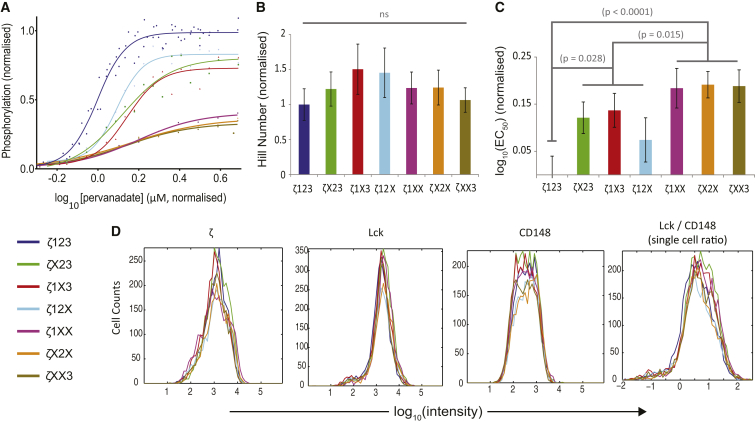
Multisite phosphorylation of the T cell receptor *ζ*-chain enhances potency but not the switchlike response. (*A*) Phosphorylation profiles of reconstituted signaling modules containing Lck, CD148, and either wild-type *ζ*-chain containing all three ITAMs (*ζ*123) or all possible combination of ITAM mutations that replace the two ITAM tyrosines with phenylalanine. A Hill function is fit to all curves to produce estimates of the (*B*) the Hill number and (*C*) potency (*EC*_50_). (*D*) Expression of *ζ*-chain, Lck, CD148, and the ratio of Lck to CD148 at the single cell level is comparable for the seven reconstituted signaling modules (see Fig. S4 for comparison of mean expression and percent positive). Representative data (*A* and *D*) and averaged parameters (*B* and *C*) are normalized to the index module (Lck, CD148, and *ζ*123) with error bars indicating mean ± SE. See Materials and Methods for details on normalization and statistical analysis. To see this figure in color, go online.

To determine the effects of multiple phosphorylation sites, we reconstituted signaling modules where the number of *ζ*-chain ITAMs is modified. We reconstituted Lck, CD148, and either the wild-type TCR *ζ*-chain, *ζ*-chain with one ITAM mutated (*ζ*X23, *ζ*1X3, and *ζ*12X), or *ζ*-chain with two ITAMs mutated (*ζ*1XX, *ζ*X2X, and *ζ*XX3), where *X* indicates that the two ITAM tyrosines have been mutated to phenylalanine (Fig. 2 *A*).

As expected, a reduced ITAM number led to a reduced maximum phosphorylation by ∼1/3 per mutated ITAM. The observation that the maximum phosphorylation depends on the number of ITAMs, but not their identity, suggests that a strict sequential mechanism of phosphorylation is unlikely to be operating. Rather, it is more likely that *ζ*-chain phosphorylation follows an unstructured random modification mechanism.

To determine the effects of multisite phosphorylation on potency and the switchlike response, we fit a Hill function to the phosphorylation profile to determine *EC*_50_ (potency) and the Hill number (switchlike response). We found no significant difference in the Hill number between the wild-type *ζ*-chain, which as a dimer contains 12 phosphorylation sites, and *ζ*-chains with ITAM mutations (Fig. 2 *B*). This observation is also consistent with a random mechanism of phosphorylation because, as discussed in the introduction, a sequential mechanism predicts that the switchlike response will be modulated by the number of sites (Fig. S1).

While the advantage of the cellular reconstitution system used here is that it maintains a normal cellular environment, a key drawback is that chemical inhibitors must be used to perturb the kinase-to-phosphatase balance. We have observed absolute Hill numbers near 4 but we are unable to rule out the possibility that these large Hill numbers were a result of a nonlinear relationship between the concentration of inhibitor and the concentration of active phosphatase. Therefore, we confined our analysis to changes in the Hill number (and other parameters) by reporting normalized parameter values as the number of phosphorylation sites is modified (see Materials and Methods).

We observed a positive correlation between receptor potency and the number of sites (Fig. 2 *C*). This effect is not predicted by the standard models of multisite phosphorylation where potency often remains unchanged as the number of sites changes (Fig. S1). A simple (and trivial) explanation for the modification in potency is that Lck and/or CD148 have different binding affinities or catalytic rates for each ITAM. However, this explanation can be ruled out by the observation that the phosphorylation profile of the three *ζ*-chain constructs with only a single intact ITAM (*ζ*1XX, *ζ*X2X, and *ζ*XX3) is similar, implying that both enzymes are equally efficient at modifying each of the three ITAMs. We conclude that it is the juxtaposition of multiple phosphorylation sites (or ITAMs) in the wild-type *ζ*-chain configuration that elicits a cooperative effect that increases potency.

To rule out that the differences in the phosphorylation profiles are a result of differences in molecular expression, we performed flow cytometry for every reconstituted signaling module. We found that the percent of cells positive for the three components and the expression levels of the three components in the seven reconstituted modules were similar (Figs. 2 *D* and S4). Moreover, we did not find any significant correlations between expression level and parameters extracted from the phosphorylation profiles (not shown). We also found that the phosphorylation kinetics is similar for ITAM mutants (Fig. S3).

Given that Lck contains an SH2 domain that can interact directly with phosphorylated sites on the *ζ*-chain, we wondered whether this domain could explain the observed changes in potency. This recruitment could increase the local concentration of Lck in the vicinity of the *ζ*-chain leading to enhanced efficiency of phosphorylation, which would be greatest for wild-type *ζ* that contains the largest number of sites. We introduced a point mutation to the Lck SH2 domain that is known to abolish binding (Lck-R154K) and performed the reconstitution experiments on all seven *ζ*-chain mutants. We found that the observed changes in potency persisted, suggesting that the SH2 domain of Lck is not responsible for increased potency (Fig. S5).

### ZAP-70 modulates T cell receptor *ζ*-chain potency but not the switchlike response

We have previously used a mathematical model to show that ZAP-70 can produce increases in both potency and the switchlike response of the T cell receptor *ζ*-chain (14). The predicted increase in the switchlike response was critically dependent on sequential phosphorylation and on ZAP-70 binding to the three *ζ*-chain ITAMs with different affinities, as previously reported in Love and Hayes (20) and Isakov et al. (31). We therefore assessed the effects of ZAP-70 on multisite *ζ*-chain phosphorylation.

We first compared the effects of ZAP-70 on the wild-type *ζ*-chain by reconstituting Lck, CD148, and *ζ*-chain with either wild-type ZAP-70 or a low activity ZAP-70 with inactivating point mutations to the SH2 and catalytic domain(s) (ZAP-70^∗^). These two domains are known to favor phosphorylation; the SH2 domains can bind with high affinity to phosphorylated ITAMs protecting them from dephosphorylation and the catalytic domain may be important to directly phosphorylate ITAM tyrosines. We compare ZAP-70 with ZAP-70^∗^ to control for the total protein load imposed on the HEK293 cells by reconstitution.

**Figure 3:**
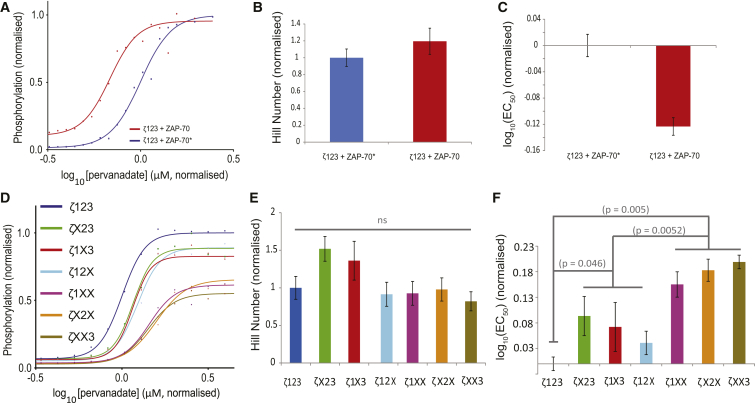
ZAP-70 enhances potency but not the switchlike response of *ζ*-chain phosphorylation. (*A*–*C*) Phosphorylation profiles of reconstituted signaling modules containing Lck, CD148, *ζ*-chain, and either wild-type ZAP-70 or ZAP-70^∗^ that contains point mutations to abolish SH2 domain binding and tyrosine kinase function. (*D*–*F*) Phosphorylation profiles of reconstituted signaling modules containing Lck, CD148, ZAP-70, and either wild-type *ζ*-chain or all possible combinations of ITAM mutations. Component expression is comparable in all signaling modules (Figs. S6 and S7). Representative data (*A* and *D*) and averaged parameters (*B*, *C*, *E*, and *F*) are normalized to an index module (Lck, CD148, *ζ*123, and ZAP-70) with error bars indicating mean ± SE. See Materials and Methods for statistical analysis. To see this figure in color, go online.

Comparing the phosphorylation profiles of ZAP-70 and ZAP-70^∗^ revealed the expected increase in potency but no change to the switchlike response was observed (Fig. 3, *A–C*), which is consistent with a recent report from Hui and Vale (32). This result is also consistent with our previous theoretical prediction that ZAP-70 will not enhance the switchlike response if the *ζ*-chain is modified by a nonsequential random mechanism (14).

We next examined the contribution of each ITAM to the ZAP-70-mediated increase in potency observed for the wild-type *ζ*-chain. We reconstituted Lck, CD148, and ZAP-70, with all *ζ*-chain variants (Fig. 3, *D*–*F*) and observed, as before, no trend in the Hill number but a significant increase in potency with the number of phosphorylation sites. Importantly, we did not observe any differences in potency within the *ζ*-chain group that contained two ITAMs or within the *ζ*-chain group that contained one ITAM, suggesting that ZAP-70 binds all *ζ*-chain ITAMs in vivo with similar affinities. As before, we examined the expression of all components to ensure that any differences in the phosphorylation profiles were not a result of differences in molecular expression (Figs. S6 and S7).

In summary, ZAP-70 enhances the phosphorylation potency of the TCR *ζ*-chain but does not alter the switchlike response. Moreover, the correlation between the potency and the number of phosphorylation sites observed in the absence of ZAP-70 is maintained in its presence.

### Phosphorylation-dependent binding rates are sufficient to explain the modulation of potency by multisite phosphorylation

We have found a correlation between the number of phosphorylation sites or ITAMs with receptor potency that cannot be explained by standard multisite phosphorylation models (see Discussion). Moreover, the observation that the group of three single (*ζ*X) and three double (*ζ*XX) ITAM mutant *ζ*-chains exhibited similar profiles indicates that Lck and CD148 modify each ITAM with similar efficiencies ruling out a trivial explanation for the enhanced potency. We therefore hypothesized that the number of phosphorylated ITAMs, independent of their specific identity, determines the rate of enzymatic modification.

**Figure 4:**
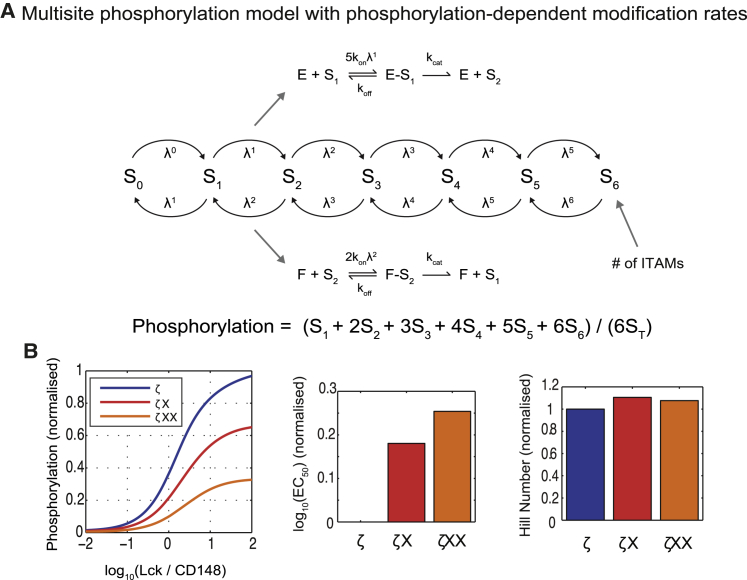
A phosphorylation-dependent enhancement in enzymatic efficiencies is sufficient to explain experimental results. (*A*) Multisite phosphorylation model showing the transitions between the concentration of phosphorylated substrate on the indicated number of ITAMs that is mediated by the kinase (*E*) and the phosphatase (shown as *F*). The model includes a fold-enhancement in the on-rate (*λ*) that is proportional to the number of phosphorylated ITAMs with the maximum increase *λ*_max_ ∼ *λ*^6^. (*B*) Phosphorylation profiles for a 6-ITAM (*ζ*), 4-ITAM (*ζ*X), and a 2-ITAM (*ζ*XX) substrate calculated using *λ* = 3 (*λ*_max_ = 729) showing the potency and Hill numbers. See the Supporting Material for computational details. To see this figure in color, go online.

We formulated a mathematical model that includes the six ITAMs on the *ζ*-chain dimer (Fig. 4 *A*). Because we do not have data relating to the effect of individual tyrosines in an ITAM we have coarse-grained the two tyrosines of each ITAM into an effective single site. We model a phosphorylation-dependent enhancement (or priming) of enzymatic efficiencies by enhancing *k*_on_ for both enzymes with increasing levels of *ζ*-chain phosphorylation. To do so, we introduce a new parameter, *λ*, which is the fold increase in the on-rate per ITAM phosphorylation so that after all ITAMs are phosphorylated the on-rate is increased by *λ*_max_ = *λ*^6^. Because all calculations are in the steady state, we found similar results if enhancement is applied to *k*_off_ or *k*_cat_ (not shown).

Computations with the mathematical model for *λ* = 3 (or *λ*_max_ = 729) produced changes in potency without large differences in the Hill number (Fig. 4 *B*). The value of *λ* that can reproduce these results was not unique because a parameter scan revealed a region of *λ* − *k*_on_ space that is consistent with changes in potency without changes in the Hill number (Fig. S8). We found that the changes in potency can be reproduced with a sufficiently large *k*_on_ when *λ* = 1, but in this limit there was residual phosphorylation that we did not observe in our experimental data (Fig. S8 *A*). Therefore, we conclude that a phosphorylation-dependent enhancement is the most likely explanation for the correlation between the number of ITAMs and receptor potency.

We next sought a plausible molecular mechanism that can be responsible for the phosphorylation-dependent enhancement. It has been previously proposed that molecular entropy may be reduced by phosphorylation of disordered amino-acid chains (33). In this model, the flexible disordered amino-acid polymer can adopt many configurations when free, but the binding of a catalytic domain reduces the number of configurations and therefore an entropic penalty to binding is incurred. This penalty can be substantially reduced if phosphorylation decreases the number of configurations of the free polymer.

**Figure 5:**
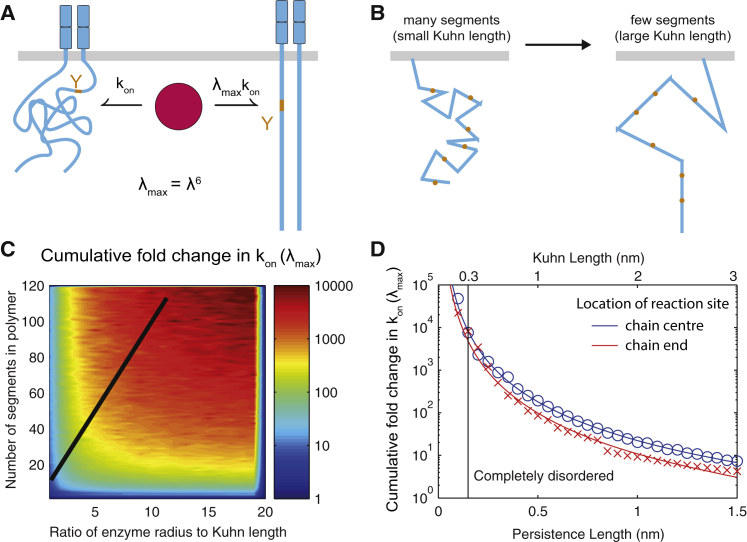
A phosphorylation-dependent enhancement in the on-rate can arise from a disorder-to-order transition. (*A*) A polymer model of the *ζ*-chain predicts that binding of the kinase (or phosphatase) is impeded by entropic disorder (*left*) and, if phosphorylation imposes local order, this impedance will be reduced when the substrate is phosphorylated with the maximal reduction occurring when the substrate is fully phosphorylated (*right*). Larger catalytic domains will incur a larger entropic penalty of binding. (*B*) The degree of disorder is determined by the number of segments whose length is known as the Kuhn length. (*C*) Heat map showing *λ*_max_ for different segment numbers (*y* axis) and for different ratios of the catalytic domain radius to Kuhn length (*x* axis). (*D*) Comparison of maximal enhancement over the Kuhn length (or persistence length) when the enzymatic binding site is at the center of the *ζ*-chain (*blue*) or at the membrane-distal tyrosine at position 152 (*red*). A Kuhn length of ∼0.5 or ∼2 amino acids corresponds to *λ*_max_ ∼ 700. Black line in (*C*) corresponds to parameter range in (*D*). See Materials and Methods for computational details. To see this figure in color, go online.

Given that the dimeric *ζ*-chain is predicted to be disordered based on the sequence of its cytoplasmic chain (determined using established predictions algorithms (34, 35)), we used a polymer model to determine an upper bound on the maximum fold-increase in the on-rate (*λ*_max_). The model includes the *ζ*-chain dimer anchored to the plasma membrane with each polymer in the dimer, modeled as a freely jointed chain with variable number of segments but a fixed overall length. The length of each segment is known as the Kuhn length (36). To calculate an upper bound for *λ*_max_, we compare the probability that the catalytic domain of an enzyme can bind the fully dephosphorylated *ζ*-chain (maximum disorder) to a hypothetical situation where the *ζ*-chain is completely ordered (i.e., exists in a single state where there is no entropic penalty to bind) as a result of maximum phosphorylation (Fig. 5 *A*).

There are two factors that determine *λ*_max_ in the model: the catalytic domain size and the Kuhn length. Larger catalytic domains will incur a larger entropic penalty (increasing *λ*_max_) and smaller Kuhn lengths increase the basal number of configurations (increasing *λ*_max_) (Fig. 5, *A* and *B*). We therefore generated a heat map showing *λ*_max_ when these parameters are varied (Fig. 5 *C*) and also show a specific slice of the heat map for clarity (Fig. 5 *D*). When the Kuhn length is 0.3 nm (or approximately the length of the *C*_*α*_–*C*_*α*_ bond), we find that *λ*_max_ ≈ 8000; whereas *λ*_max_ ≈ 700 (used in the phosphorylation model above) can be found when the Kuhn length is 0.5 nm, which is consistent with the flexibility previously reported for polypeptide chains (37).

These calculations are based on the enzyme catalytic domain interacting with a test tyrosine at the center of the chain, but we have also performed the calculations with a test tyrosine at position 152, which is the most distal tyrosine for murine *ζ*-chain, finding only a weak dependence on *λ*_max_ (Fig. 5 *D*).

In summary, a phosphorylation-dependent enhancement in the binding interactions can produce the observed correlation between the number of phosphorylation sites and receptor potency, and this enhancement is consistent with a disorder-to-order transition upon phosphorylation that reduces the molecular entropic penalty to binding.

## Discussion

Using cellular reconstitution, we have performed systematic experiments to test predictions of mathematical models for multisite phosphorylation as applied to the T cell receptor *ζ*-chain. In contrast to model predictions, we have found no evidence that multiple phosphorylation sites are able to modulate the switchlike response but have found that multiple sites can modulate receptor potency and maximum phosphorylation.

Reconstitution is a powerful reductionist method that enables the study of a well-defined signaling module without the feedbacks and unknown interactions that occur in native cells. Hui and Vale (32) reconstituted the TCR *ζ*-chain with different concentrations of Lck and CD45 on liposomes using purified proteins and found only a modest Hill number of 2 that did not change when ZAP-70 was included, which is consistent with our finding. James and Vale (30) reconstituted the TCR and a number of other TCR proximal components in HEK293 cells to investigate the mechanism by which ligand binding to the TCR induces intracellular phosphorylation, a process termed “TCR triggering” (16). In contrast to their work, we did not find detectable basal phosphorylation when reconstituting the TCR, Lck, and CD148 alone in HEK293 cells. This discrepancy is likely a result of the high expression of our extracellular domain truncated phosphatase (38).

This work has several implications for TCR proximal signaling. We have previously used a mathematical model to show that multiple *ζ*-chain ITAMs, their sequential phosphorylation, and differences in ZAP-70 binding affinities can generate emergent switchlike responses (14). However, we found no evidence for sequential phosphorylation of the TCR *ζ*-chain in this work. Two previous studies have provided evidence for sequential phosphorylation but differed in the reported sequence (23, 24). The discrepancy with this work may be explained by the fact that the previous studies relied on measurements without systematic variations to kinase/phosphatase. Moreover, we found no evidence for in vivo differences in ZAP-70 binding affinities in this work because, for example, the potencies of *ζ*-chains with single intact ITAMs were identical in the presence of ZAP-70 (Fig. 3 *F*). Previous work has suggested differences in ZAP-70 binding affinities ranging from 3- to 30-fold based on measurements with the isolated tandem SH2 domains (31, 39) but a more recent study has shown markedly different behavior of the full protein (40). We have found that ZAP-70 increases receptor potency but not the switchlike response consistent with a previous study by Hui and Vale (32). The combination of random phosphorylation and similar ZAP-70 binding affinities predicts that multiple ITAMs will not contribute to a switchlike response (see Fig. 5A in Mukhopadhyay et al. (14)), which is the observation made in this work.

What is then the purpose of multiple ITAMs in the TCR complex? Although the TCR *ζ*-chain ITAMs are not sequentially phosphorylated, they may be involved in kinetic proofreading, which relies on a delay (that can be produced by either distributive sequential or distributive random phosphorylation) between initial ligand binding and downstream signaling for antigen discrimination (18, 20, 41, 42, 43, 44, 45, 46). The effect with the largest magnitude is the intuitive difference in the maximum phosphorylation when ITAMs are removed. This likely reflects the signal amplification property of the TCR and is consistent with previous work showing that, for example, T cell proliferation scales with the number, but not the identity, of individual ITAMs (47) and more recent work showing that the *ζ*-chain is dispensable for many T cell responses (48). This leaves open the question of why there are highly conserved differences in individual ITAMs within the TCR (see, for example, Fig. S1 in Mukhopadhyay et al. (14) for an alignment).

The observation that phosphorylation of one ITAM can enhance the phosphorylation of other ITAMs is related to the phenomenon termed “phosphorylation priming”. Phosphoproteomic analysis revealed that phosphorylation of one site can enhance the phosphorylation of neighboring sites within ∼10 amino acids (49). Moreover, recent studies have found evidence for such phosphorylation priming in an actin cross-linking protein (50) and within the two tyrosines of the ITAM (51).

The mechanism underlying phosphorylation priming is incompletely understood. A possible mechanism is a disorder-to-order transition that phosphorylation may induce in intrinsically disordered regions of proteins (33). The T cell receptor *ζ*-chain, and indeed many noncatalytic tyrosine-phosphorylated receptors (52), have cytoplasmic tails that are thought to be intrinsically disordered with a continuum of states that can exhibit high molecular entropy. The binding of a modifying enzyme to a disordered substrate incurs an entropic penalty because binding is often compatible with only a subset of all states. If phosphorylation induces local order, it may increase the effective on-rate for substrate binding. We note that a switchlike response is not introduced by this mechanism if it applies equally to both modifying enzymes as we have implemented. But a switchlike response can be generated if, for example, the enhancement applies selectively to one modifying enzyme or if the reduced entropic binding allows the substrate to be sequestered away from both modifying enzymes (6, 7, 13).

Another mechanism that may explain phosphorylation priming across ITAMs is the electrostatically mediated association of the cytoplasmic tails of transmembrane receptors with the membrane. Such interactions have been demonstrated for the CD3*E* subunit of the TCR complex (53, 54), the TCR *ζ*-chain (25), and the ITIM-containing receptor PECAM-1 (55). Because multisite phosphorylation drastically alters the charge of molecules it may induce dissociation of the cytoplasmic tails from the membrane. This mechanism can induce phosphorylation priming if the cytoplasmic tail becomes more accessible to the modifying enzymes when dissociated from the membrane. We note that published models of multisite phosphorylation have often reported switchlike responses, but not changes in potency (8, 9, 10, 11, 12, 13, 14, 15). Our previous work has shown that local rebinding can lead to switchlike response when diffusion is limited, but we did not observe a change in potency (12).

Previous experimental work has reported switchlike responses by multisite phosphorylation that can apply to unstructured proteins such as the TCR *ζ*-chain. For example, phosphorylation has been shown to generate a switchlike response in the membrane localization of the scaffold Ste5 by bulk electrostatics, whereby phosphorylation increases the negative charge of the substrate and therefore leads to reduced membrane association (56, 57). As discussed above, the association of the TCR *ζ*-chain with the membrane is thought to be regulated by bulk electrostatics (25, 53). A key difference between Ste5 and the TCR is that bulk electrostatics can completely alter the localization of Ste5 whereas bulk electrostatics may only modestly alter the average distance of the TCR cytoplasmic tails from the membrane.

Although frequently responsible for switchlike responses in other proteins, we have found that multisite phosphorylation of the TCR *ζ*-chain does not modulate the switchlike response but can modulate the potency and maximum phosphorylation of the receptor. A key difference between the TCR and other multisite substrates known to exhibit switchlike responses is that phosphorylation sites in the latter case are thought to act in concert. For example, in the case of Ste5, Sic1, and NFAT, multiple phosphorylation sites collectively regulate association with the membrane (56, 57), association with another protein (58), or a conformational change (59, 60), respectively. In contrast, the 10 ITAMs on the TCR can each independently provide docking sites for the effector kinase ZAP-70, which can propagate downstream signaling (20). In this way the TCR, and possibly other immune receptors, have acquired multiple phosphorylation sites that may individually act to amplify ligand binding events.

## Author Contributions

H.M., B.d.W., P.A.v.d.M., and O.D. designed research; H.M., B.d.W., L.C., J.A., and O.D. performed research; H.M., B.d.W., L.C., J.A., P.K.M., P.A.v.d.M., and O.D. analyzed data; and H.M., B.d.W., P.A.v.d.M., and O.D. wrote the article.

## Supporting Citations

References (61, 62, 63, 64, 65) appear in the Supporting Material.

## Supporting Material

Document S1. Supporting Materials and Methods, Figs. S1–S8, and Tables S1–S3

Document S2. Article plus Supporting Material
