# Supplementary material for: Multisite Phosphorylation Modulates the T Cell Receptor ζ-Chain Potency but not the Switchlike Response
Source: Biophys J. 2016 Apr 26;110(8):1896–906. doi: 10.1016/j.bpj.2016.03.024 (PMC4850346; doi:10.1016/j.bpj.2016.03.024)
Supplement: Document S1. Supporting Materials and Methods, Figs. S1–S8, and Tables S1–S3 [file mmc1.pdf]

Biophysical Journal, Volume *110*

Supplemental Information

Multisite Phosphorylation Modulates the T Cell Receptor ζ-Chain Potency but not the Switchlike Response

Himadri Mukhopadhyay, Ben de Wet, Lara Clemens, Philip K. Maini, Jun Allard, P. Anton van der Merwe, and Omer Dushek

Supporting Material: Multisite Phosphorylation Modulates T cell Receptor ζ-chain Potency but not the Switch-like Response

Himadri Mukhopadhyay[†*], Ben de Wet[†*], Lara Clemens[^], Philip K. Maini[‡], Jun Allard[^], P. Anton van der Merwe[†,¶], Omer Dushek[†,‡,¶]

[†]Sir William Dunn School of Pathology, University of Oxford, Oxford, Oxfordshire, United Kingdom
[‡]Wolfson Centre for Mathematical Biology, Mathematical Institute, University of Oxford, Oxford, Oxfordshire, United Kingdom
[^]Department of Mathematics, University of California, Irvine, United States
[*] These authors contributed equally to this work
[¶]Corresponding authors

Supporting Text

Mathematical model of multisite phosphorylation

The mathematical model includes a multisite substrate (S, TCR ζ-chain) that is phosphorylated by a kinase (E, Lck) and dephosphorylated by a phosphatase (F, CD148) by an unstructured random mechanism. We coarse-grained the tyrosines in each of the 6 ITAMs present in the ζ-chain dimer into a single effective site. Each elementary modification reaction is governed by an on-rate (k_{on}), an off-rate (k_{off}), and by a catalytic rate (k_{cat}),

$$\begin{aligned} E + S_j \underset{k_{\text{off}}}{\overset{(N-j)k_{\text{on}}\lambda^j}{\rightleftharpoons}} ES_j \overset{k_{\text{cat}}}{\rightarrow} E + S_{j+1} \\ F + S_j \underset{k_{\text{off}}}{\overset{jk_{\text{on}}\lambda^j}{\rightleftharpoons}} FS_j \overset{k_{\text{cat}}}{\rightarrow} F + S_{j-1} \end{aligned} \tag{1}$$

where N is the number of ITAMs ($N = 6$ in this case) and S_j is the concentration of substrate phosphorylated on j ITAMs. We include a phosphorylation-dependent enhancement in the enzymatic affinity by increasing k_{on} by a factor of λ for each phosphorylated site for both enzymes. The normalised total phosphorylation of the substrate was calculated at steady-state as follows,

$$\text{Phosphorylation} = \sum_{j=0}^{N} j(S_j + ES_j + FS_j)/(6S_T)$$

where S_T is the total concentration of substrate. We assume for all calculations that the reaction rates are identical for both modifying enzymes.

The system of ordinary-differential-equations (ODEs) representing the biochemical reaction network based on this scheme was generated in BioNetGen (1) and integrated in Matlab (Mathworks, MA). The parameters used are $k_{\text{off}} = 1$ s^{-1}, $k_{\text{cat}} = 10$ s^{-1}, and $S_T = 1$ μm^{-2} with the indicated variation to k_{on} (μm^{-2}s^{-1}) and λ (dimensionless). The ratio of E to F was varied by varying F over a 10^6-fold range to obtain the dose-response.

Mathematical model of polymer configurations

Polymer model for the TCR ζ-chain. Disordered chains of amino acids are often modeled as freely-jointed chains as these models agree favourably with experiments (2–4). In these models, each amino acid is assumed to be a rigid rod of length $\delta = 0.3$ nm, referred to as the Kuhn length, connected by a joint that freely explores configurations in three dimensions. The persistence length, an alternative quantity measuring protein flexibility, is equal to half the Kuhn length (5).

The murine ζ-chain has 113 amino acids in its cytoplasmic tails with tyrosines at positions 72/83 (ζ1 ITAM), 111/123 (ζ2 ITAM), and 142/153 (ζ3 ITAM). Assuming a standard disordered chain leads to $N = 113$ segments with individual segment lengths (or Kuhn lengths) of $\delta = 0.3$ nm. To explore the possibility that the chain has some ordered structure, we assume that the contour length $L_c = \delta N \approx 33.9$ nm is fixed but explore a range of Kuhn lengths. This ordered structure could arise because of inter-residue interactions in the native unphosphorylated state or following post-translational modification, such as tyrosine phosphorylation. We note that in these calculations phosphorylation of a site on one ζ-chain in the dimer only induces local order and does not induce ordering of the other chain.

To model interaction of the ζ-chain with its enzymes, we estimate the geometric properties of the catalytic domains of Lck and CD148. The volume of the catalytic domain of Lck is 45 nm^3 based on its crystal structure. Alternatively, the volume can be estimated based on an estimate for protein density, which is approximately 1.41g / cm^3. Using this value and the fact that the catalytic domain consists of 254 amino acids whose molecular mass is 29 kDa, we estimate the volume as follows,

$$(29 \times 1000\text{Da}) * (1.66 \times 10^{-27}\text{kg / Da}) * (1000\text{g / kg})/(1.41\text{g / cm}^3) = 34\text{nm}^3. \tag{2}$$

This estimate is similar to the estimate based on the crystal structure. Approximating the domain as a sphere, we arrive at a radius $r_K \approx 3.2 - 3.5$nm. Given that the catalytic domain of CD148 is similar in size, we assume for simplicity that it has similar dimensions.

Calculation of occlusion probability. We perform Monte Carlo simulation on the polymer model described above using the Metropolis algorithm, which generates an ensemble of polymer configurations at constant temperature (6). At each configuration in the ensemble we determine whether the enzyme is occluded from its binding site, which occurs with probability P. The binding site represents a tyrosine that is to be modified at a specific location along the length of the polymer.

From the thermodynamic detailed balance condition (6), the dissociation constants $K \equiv k_{\text{off}}/k_{\text{on}}$ of the floppy (native) state (K_F) and the rigid (partially phosphorylated) state (K_R) are related by

$$\frac{K_R}{K_F} = \exp\left(\frac{G_R - G_F}{k_B T}\right) \tag{3}$$

$$= \exp\left(\frac{S_F - S_R}{k_B}\right) \tag{4}$$

$$= \frac{P_F}{P_R} \tag{5}$$

where $G_j = E_j - TS_j$ is the free energy of binding in the floppy or rigid state, $S_j = k_B \ln P_j$ is the molecular entropy of binding, and P_j is the probability in the canonical ensemble that the configuration allows for binding.

To estimate the maximum enhancement in binding (λ_{max}) we compare the probability of binding in the floppy state to the probability of binding when the ζ-chain polymer is perfectly rigid (i.e. with $N = 1$ segments whose Kuhn length is $\delta = L_c = 33.9$ nm). In this latter limit the enzyme is never occluded by the rest of the polymer (or the other polymer in the dimer) and therefore $P_R = 1$. It follows that λ_{max} can be calculated based on P_F as follows,

$$\lambda_{\text{max}} = \frac{K_F}{K_R} = 1/P_F. \tag{6}$$

Polymer model variants. The binding enhancement depends on the location of binding site along the polymer. We find that λ_{max} is maximal for binding sites near the midpoint of the polymer $i = N/2$ with a weak dependence on binding sites that are distal to the midpoint (Fig. 5D). We note that binding sites in the simulations are located at segment joints leading to apparent discontinuities (Fig. 5D).

The polymer simulations were performed in free space but since the TCR ζ-chain is a transmembrane protein, we anticipated that an interaction with the membrane would alter the number of available configurations and therefore our estimates of λ_{max}. Therefore, we modified the simulation to include a two-dimensional plane with the TCR ζ-chain polymer embedded therein and stipulated that neither the polymer nor the enzyme are able to cross the plane (membrane). Surprisingly, we find a weak dependence on the presence of the membrane that results from two competing effects (not shown). First, the membrane leads to more configurations in which the enzyme is sterically blocked from its site (increasing λ_{max}). Second, the polymer tends to straighten out when constrained by a plane (7) (decreasing λ_{max}). The net effect is a weak dependence on the presence of a membrane.

BioNetGen Script

```
# A substrate with six ITAMs sites is modified by a kinase (E) and a phosphatase (F).
# Modifications follow a random phosphorylation scheme.

begin parameters

# Parameter 'place holders' are defined in BioNetGen, and these are modified in MATLAB
# to include, for example, phosphorylation-dependent on-rates (Fig . 4A)

# kinase on rates for each ITAM

ek_on1 1
ek_on2 1
ek_on3 1
ek_on4 1
ek_on5 1
ek_on6 1

# phosphatase on rates for each ITAM

fk_on1 1
fk_on2 1
fk_on3 1
fk_on4 1
fk_on5 1
fk_on6 1
```

```
# kinase off rates for each ITAM

ek_off1 1
ek_off2 1
ek_off3 1
ek_off4 1
ek_off5 1
ek_off6 1

# phosphatase off rates for each ITAM

fk_off1 1
fk_off2 1
fk_off3 1
fk_off4 1
fk_off5 1
fk_off6 1

# kinase rates of catalysis

ek_cat1 1
ek_cat2 1
ek_cat3 1
ek_cat4 1
ek_cat5 1
ek_cat6 1

# phosphatase rates of catalysis

fk_cat1 1
fk_cat2 1
fk_cat3 1
fk_cat4 1
fk_cat5 1
fk_cat6 1

# total amounts of substrate, kinase and phosphatase

S_T 100
E_T 1e3
F_T 1e3

end parameters

# Define molecule types.
# Multisite substrate (S) can be fully dephosphorylated (U), phosphorylated on one ITAM (P),
# phosphorylated on two ITAMs (2P), etc.
# Only catalytic domains are defined for the kinase (ecat) and phosphatase (fcat).

begin molecule types

S(Y~U~P~2P~3P~4P~5P~6P)
E(ecat)
F(fcat)

end molecule types
```

```
# Define initial conditions.

begin seed species

S(Y~U) S_T
E(ecat) E_T
F(fcat) F_T

end seed species

# Define reaction network.

begin reaction rules

# fully dephosphorylated substrate <-> substrate phosphorylated on one ITAM

E(ecat) + S(Y~U) <-> E(ecat!1).S(Y~U!1) ek_on1, ek_off1
E(ecat!1).S(Y~U!1) -> E(ecat) + S(Y~P) ek_cat1

F(fcat) + S(Y~P) <-> F(fcat!1).S(Y~P!1) fk_on1, fk_off1
F(fcat!1).S(Y~P!1) -> F(fcat) + S(Y~U) fk_cat1

# substrate phosphorylated on one ITAM <-> substrate phosphorylated on two ITAMs

E(ecat) + S(Y~P) <-> E(ecat!1).S(Y~P!1) ek_on2, ek_off2
E(ecat!1).S(Y~P!1) -> E(ecat) + S(Y~2P) ek_cat2

F(fcat) + S(Y~2P) <-> F(fcat!1).S(Y~2P!1) fk_on2, fk_off2
F(fcat!1).S(Y~2P!1) -> F(fcat) + S(Y~P) fk_cat2

# substrate phosphorylated on two ITAMs <-> substrate phosphorylated on three ITAMs

E(ecat) + S(Y~2P) <-> E(ecat!1).S(Y~2P!1) ek_on3, ek_off3
E(ecat!1).S(Y~2P!1) -> E(ecat) + S(Y~3P) ek_cat3

F(fcat) + S(Y~3P) <-> F(fcat!1).S(Y~3P!1) fk_on3, fk_off3
F(fcat!1).S(Y~3P!1) -> F(fcat) + S(Y~2P) fk_cat3

# substrate phosphorylated on three ITAMs <-> substrate phosphorylated on four ITAMs

E(ecat) + S(Y~3P) <-> E(ecat!1).S(Y~3P!1) ek_on4, ek_off4
E(ecat!1).S(Y~3P!1) -> E(ecat) + S(Y~4P) ek_cat4

F(fcat) + S(Y~4P) <-> F(fcat!1).S(Y~4P!1) fk_on4, fk_off4
F(fcat!1).S(Y~4P!1) -> F(fcat) + S(Y~3P) fk_cat4

# substrate phosphorylated on four ITAMs <-> substrate phosphorylated on five ITAMs

E(ecat) + S(Y~4P) <-> E(ecat!1).S(Y~4P!1) ek_on5, ek_off5
E(ecat!1).S(Y~4P!1) -> E(ecat) + S(Y~5P) ek_cat5

F(fcat) + S(Y~5P) <-> F(fcat!1).S(Y~5P!1) fk_on5, fk_off5
F(fcat!1).S(Y~5P!1) -> F(fcat) + S(Y~4P) fk_cat5

# substrate phosphorylated on five ITAMs <-> substrate phosphorylated on six ITAMs
```

```
E(ecat) + S(Y~5P) <-> E(ecat!1).S(Y~5P!1) ek_on6, ek_off6
E(ecat!1).S(Y~5P!1) -> E(ecat) + S(Y~6P) ek_cat6

F(fcat) + S(Y~6P) <-> F(fcat!1).S(Y~6P!1) fk_on6, fk_off6
F(fcat!1).S(Y~6P!1) -> F(fcat) + S(Y~5P) fk_cat6


end reaction rules

# Define observables.

begin observables

1 Molecules Szero S(Y~U!?)
2 Molecules Sone S(Y~P!?)
3 Molecules Stwo S(Y~2P!?)
4 Molecules Sthree S(Y~3P!?)
5 Molecules Sfour S(Y~4P!?)
6 Molecules Sfive S(Y~5P!?)
7 Molecules Ssix S(Y~6P!?)

end observables


generate_network({overwrite=>1});

writeMfile({});
```

Supporting References

1. Hlavacek, W. S., J. R. Faeder, M. L. Blinov, R. G. Posner, M. Hucka, and W. Fontana, 2006. Rules for modeling signal-transduction systems. *Science's STKE : signal transduction knowledge environment* 2006:re6.

2. Reeves, D., K. Cheveralls, and J. Kondev, 2011. Regulation of biochemical reaction rates by flexible tethers. *Physical Review E - Statistical, Nonlinear, and Soft Matter Physics* 84:1–12.

3. Kutys, M. L., J. Fricks, and W. O. Hancock, 2010. Monte Carlo analysis of neck linker extension in kinesin molecular motors. *PLoS computational biology* 6:e1000980.

4. Van Valen, D., M. Haataja, and R. Phillips, 2009. Biochemistry on a leash: the roles of tether length and geometry in signal integration proteins. *Biophysical journal* 96:1275–92.

5. Andrews, S. S., 2014. Methods for modeling cytoskeletal and DNA filaments. *Physical Biology* 11:011001.

6. Boal, D., 2012. Mechanics of the Cell. Cambridge University Press.

7. Milner, S., T. Witten, and M. Cates, 1988. Theory of the grafted polymer brush. *Macromolecules* 21:2610–2619.

Supporting Tables

	Hill Number (normalised)			$\log_{10}(EC_{50})$ (normalised)		
	Mean	SEM	N	Mean	SEM	N
ζ123	1.000	0.225	5	0.000	0.040	9
ζX23	1.221	0.244	5	0.121	0.033	9
ζ1X3	1.505	0.359	5	0.137	0.036	9
ζ12X	1.454	0.354	5	0.074	0.047	9
ζ1XX	1.237	0.228	5	0.184	0.042	9
ζX2X	1.243	0.249	5	0.191	0.028	8
ζXX3	1.065	0.174	5	0.188	0.034	8

Table S1: Hill Numbers and $\log_{10}(EC_{50})$ for the data in Fig. 2

	Hill Number (normalised)			$\log_{10}(EC_{50})$ (normalised)		
	Mean	SEM	N	Mean	SEM	N
ζ123	1.000	0.152	5	0.000	0.013	5
ζX23	1.519	0.164	4	0.093	0.038	4
ζ1X3	1.361	0.258	3	0.072	0.048	3
ζ12X	0.915	0.160	3	0.041	0.023	3
ζ1XX	0.927	0.158	3	0.155	0.025	3
ζX2X	0.978	0.154	3	0.183	0.022	3
ζXX3	0.821	0.127	4	0.199	0.013	4

Table S2: Hill Numbers and $\log_{10}(EC_{50})$ for the data in Fig. 3

	Hill Number (normalised)			$\log_{10}(EC_{50})$ (normalised)		
	Mean	SEM	N	Mean	SEM	N
ζ123	1.000	0.173	4	0.000	0.023	4
ζX23	1.066	0.153	4	0.048	0.015	4
ζ1X3	1.062	0.184	4	0.032	0.034	4
ζ12X	1.097	0.238	4	0.041	0.029	4
ζ1XX	1.036	0.117	4	0.105	0.012	4
ζX2X	1.070	0.105	4	0.113	0.047	4
ζXX3	0.912	0.212	4	0.163	0.047	4

Table S3: Hill Numbers and $\log_{10}(EC_{50})$ for the data in Fig. S5

Supporting Figures

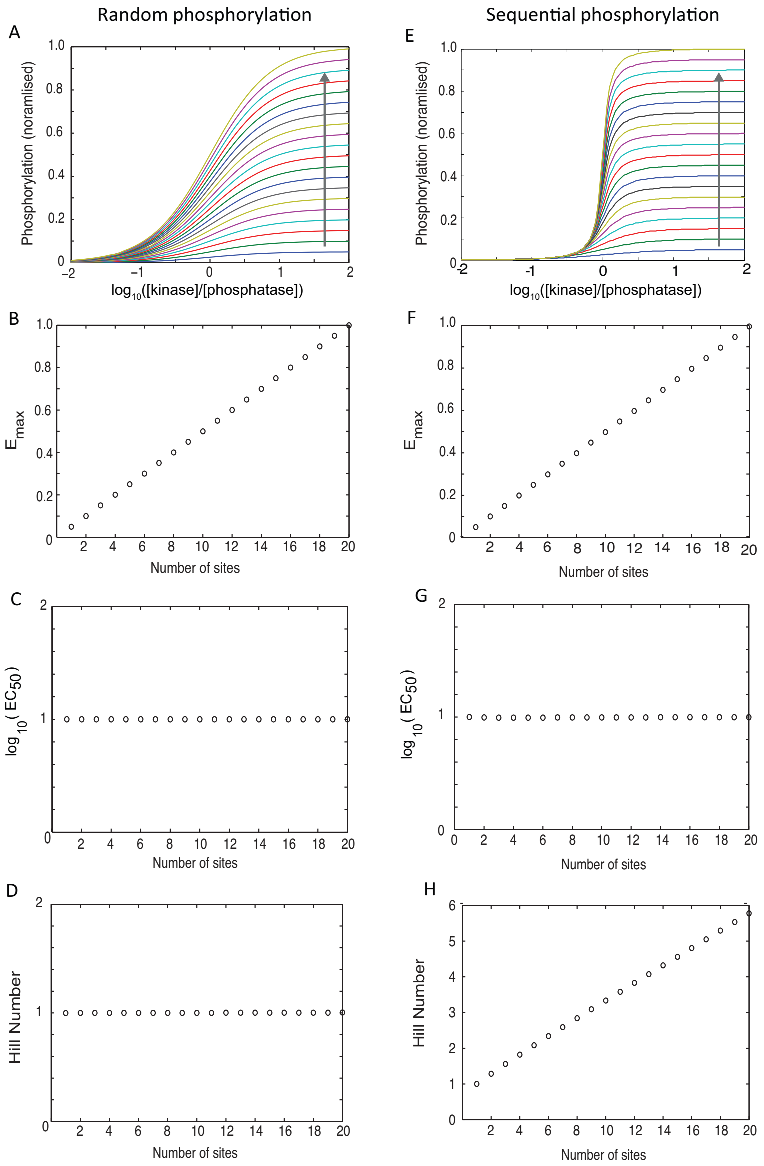

Fig S1: **Phosphorylation profiles of multisite phosphorylation based on a random and sequential phosphorylation mechanism.** Mathematical model of a substrate containing 1, 2, 3, ... 20 phosphorylation sites (arrow indicates direction of increasing number of sites) that can be phosphorylated by a kinase and dephosphorylated by a phosphatase in any order (A-D, Random phosphorylation) or in a strict sequential order (E-H, Sequential phosphorylation). Phosphorylation (y-axis in A, E) is the total phosphorylation calculated by adding the concentration of substrate phosphorylated on N sites multiplied by N normalised to the maximum phosphorylation of a 20-site substrate (see Fig. 4A). Multisite phosphorylation does not modulate potency (EC_{50}) when phosphorylation is random or sequential but does modulate the switch-like response when phosphorylation is sequential.

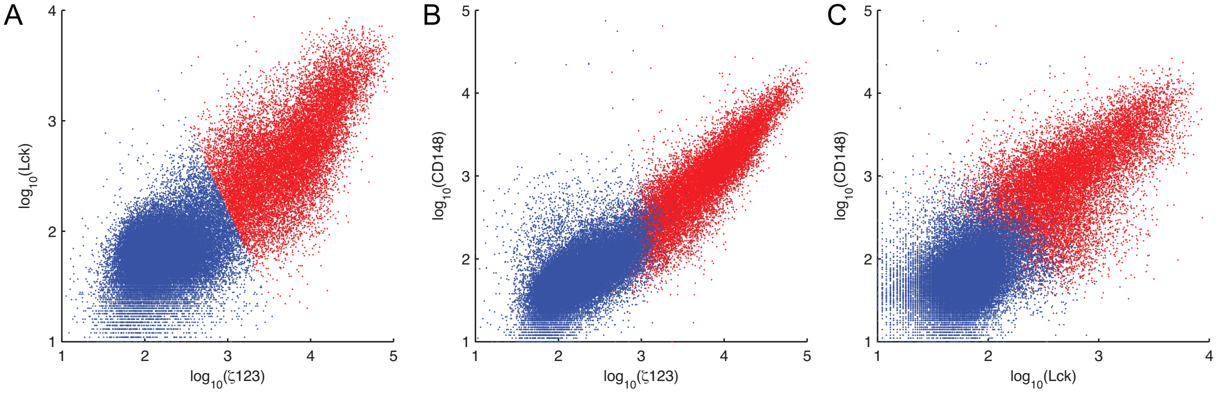

Fig S2: **Two-dimensional projections of expression data shown in Fig. 1B.** A-C) Pairwise projections of the three-dimensional molecular expression for ζ123, Lck, and CD148.

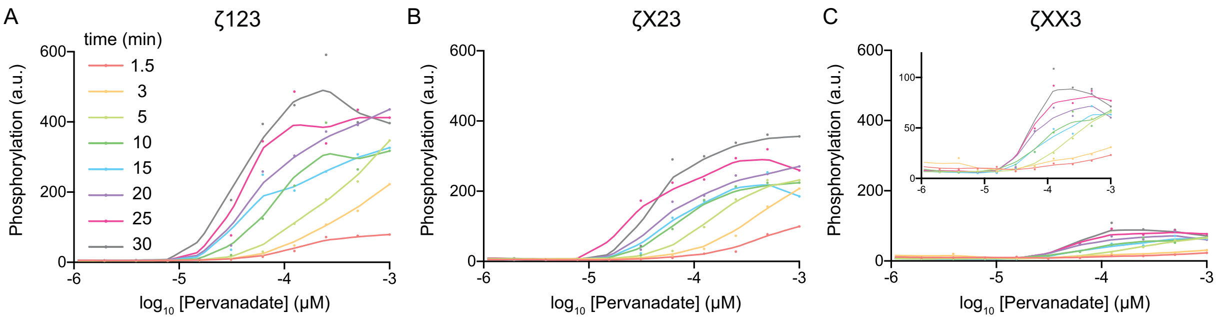

Fig S3: **Phosphorylation timecourse for modules with different number of phosphorylation sites follow similar kinetics to steady-state.** Phosphorylation for reconstitution of Lck, CD148 and A) ζ123, B) ζX23, and C) ζXX3 at the indicated time. Solid line is a running mean of the experimental data (filled circles).

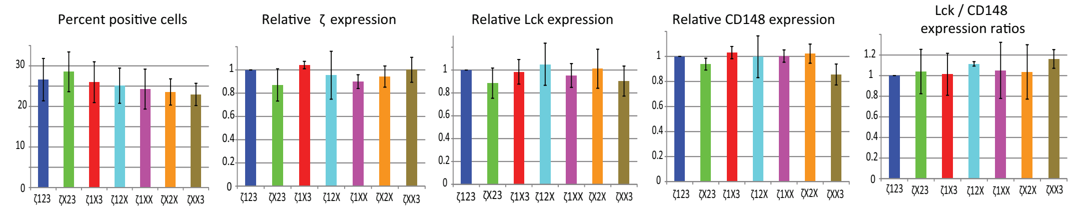

Fig S4: **Mean percent positive and mean molecular expression levels for indicated components for each reconstituted signalling module shown in Fig. 2B,C.** Expression is calculated only for the positive cells as determined by a clustering algorithm (see Fig. 1B) and normalised to the wild-type ζ-chain in each case (ζ123).

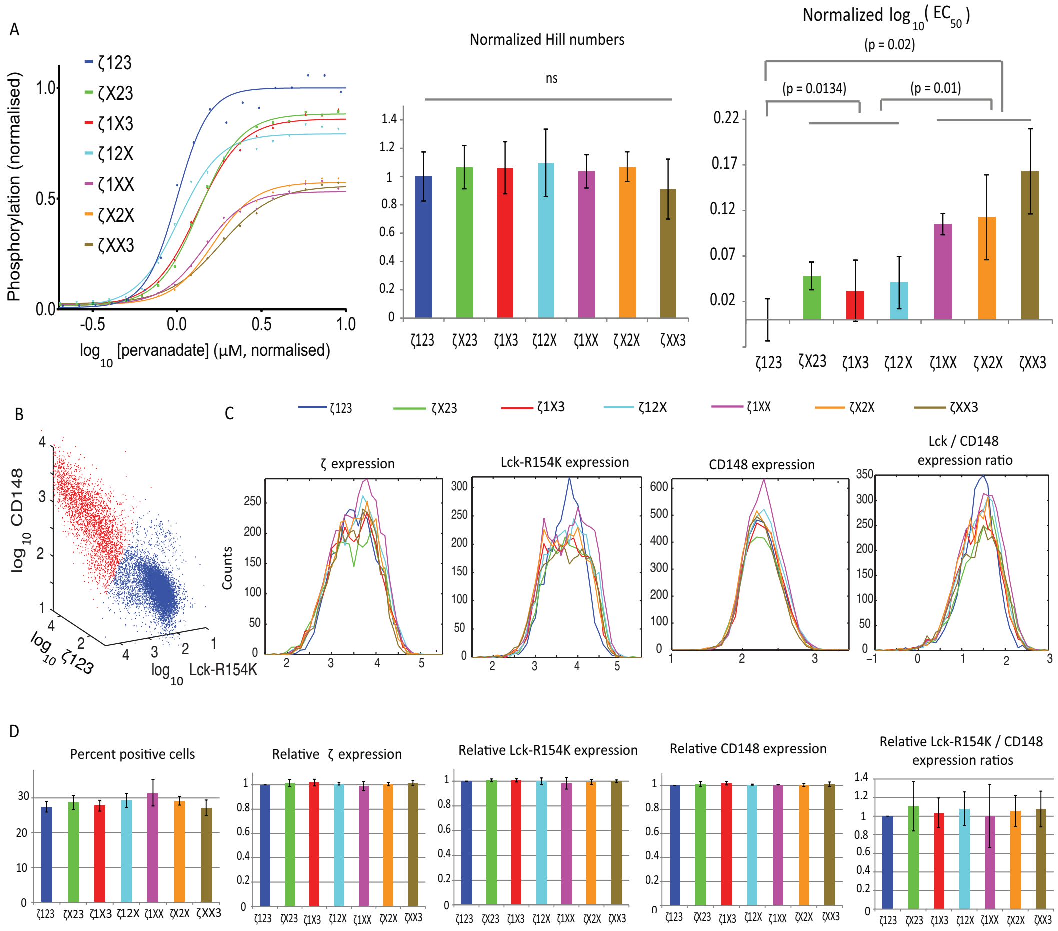

Fig S5: **Modulation of potency and not the switch-like response by multisite phosphorylation is independent of the Lck SH2 domain.** A) Phosphorylation profiles of reconstituted signalling modules containing Lck with a point mutation in the SH2 domain that abolishes binding (Lck-R154K), CD148, and either wild-type ζ-chain (ζ123) or all possible ITAM mutations as indicated. A Hill function is fit to all curves to produce estimates of the potency (EC_{50}) and the Hill number. B-D) Analysis of the expression of each component reveals comparable expression levels for each of the 7 reconstituted signalling modules. Representative clustering of cells as positive (red) or negative (blue) for expression of all 3 components along with C) representative expression profiles of each component in all 7 reconstituted signalling modules. D) Mean percent positive and mean expression of the indicated component (from the positive population) relative to the signalling module with wild-type ζ-chain (ζ123, blue). Representative data and averaged parameters are normalised to the index module (Lck-R154K, CD148, and ζ123) with error bars indicating ±sem. See Materials & Methods for details on normalisation and statistical analysis.

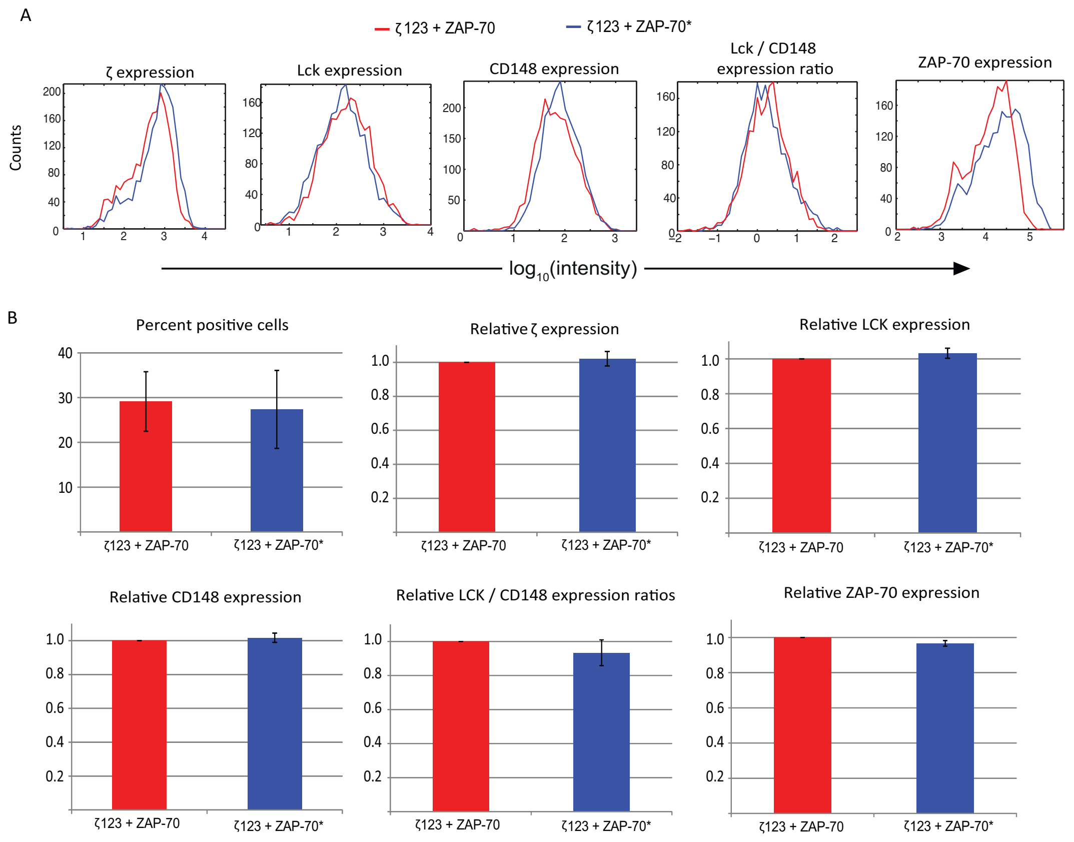

Fig S6: **Molecular expression levels for reconstituted signalling modules shown in Fig 3A-C.** A) Representative flow cytometery data showing expression of the indicated component and the ratio of Lck to CD148 in individual cells. B) Mean percent positive and mean expression of the indicated component (from the positive population) relative to the signalling module with wild-type ZAP-70 (red). The data are taken from at least 3 independent experiments and error bars indicate ±sem.

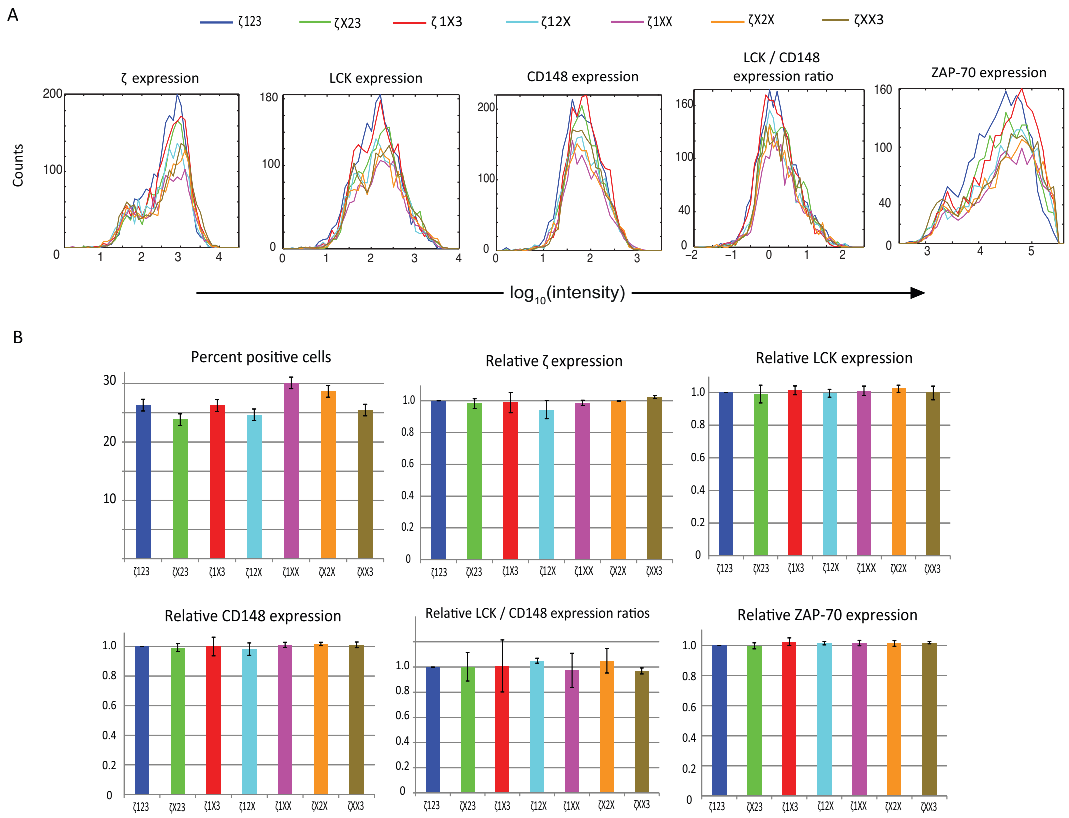

Fig S7: **Molecular expression levels for reconstituted signalling modules shown in Fig. 3D-F.** A) Representative flow cytometry data showing expression of the indicated component and the ratio of Lck to CD148 in individual cells. B) Mean percent positive and mean expression of the indicated component (from the positive population) relative to the signalling module with wild-type ζ-chain (ζ123, blue). The data are taken from at least 3 independent experiments and error bars indicate $\pm$sem.

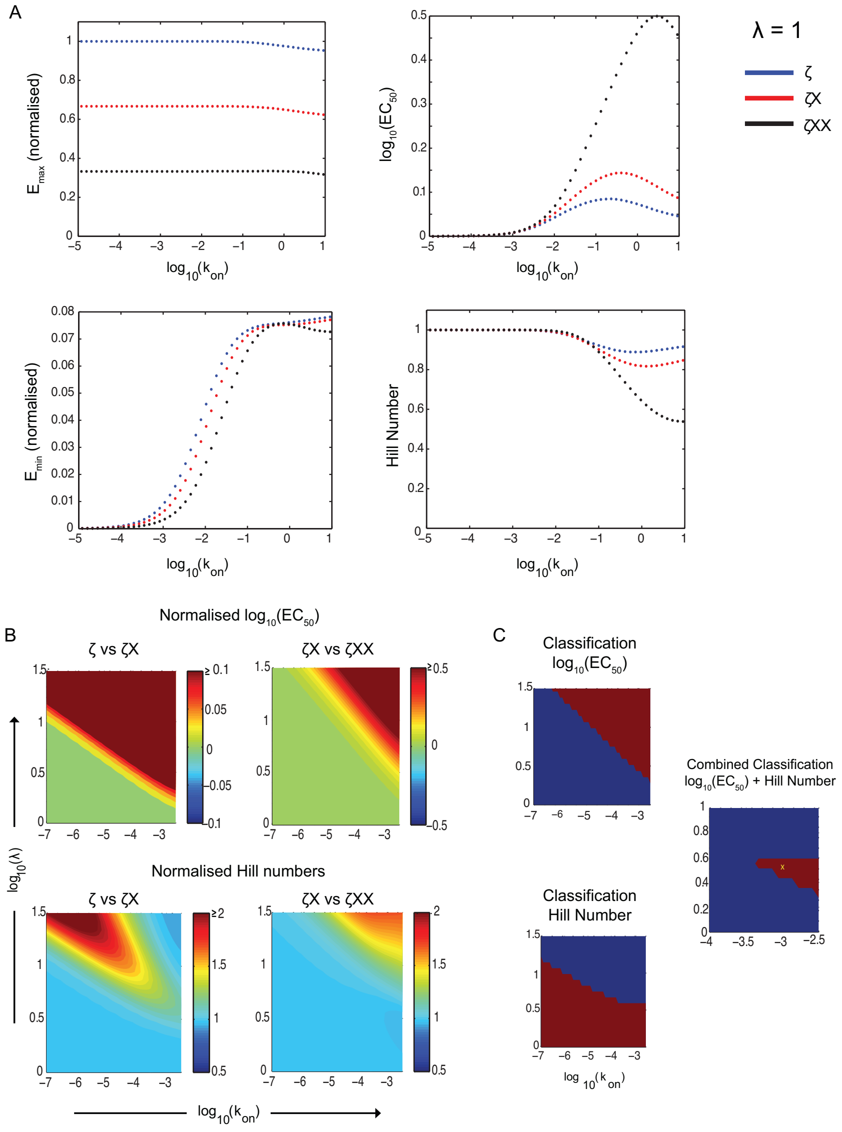

Fig S8: **Parameter scan for multisite phosphorylation model in Fig. 4A,B**. A) In the standard model without a phosphorylation-dependent enhancement ($\lambda = 1$) a change in potency can be observed when the enzymatic affinity is large ($k_{\text{on}} > 0.01\ \mu\text{m}^{-2}\text{s}^{-1}$) but in this case the large affinity of the enzymes to the substrate results in increased basal phosphorylation (E_{min}) which is not observed in the experimental data. B) Heat maps showing the difference in potency (top, on a log-scale) and the fold change in the Hill number (bottom) for different values of the enhancement factor (λ) and the affinities of the enzymes (modified by increasing k_{on}). Comparisons are shown between the 6 site (ζ) and 4 site (ζX) substrate and between the 4 site (ζX) and 2 site (ζXX) substrate. C) Binary maps of the region of $\lambda - k_{\text{on}}$ parameter space where the experimental trends are satisfied for the potency and Hill number (shown in red). These binary classifications are multiplied to obtain the overall combined region of parameter space where both the potency and Hill number trends are satisfied (X denoted the parameter values used to generate the phosphorylation profiles in Fig 4B). See Materials & Methods for computational details.
