# Supplementary material for: Multisite Phosphorylation Modulates the T Cell Receptor ζ-Chain Potency but not the Switchlike Response
Source: Biophys J. 2016 Apr 26;110(8):1896–906. doi: 10.1016/j.bpj.2016.03.024 (PMC4850346; doi:10.1016/j.bpj.2016.03.024)
Supplement: Document S2. Article plus Supporting Material [file mmc2.pdf]

Biophysical Journal
Article

Biophysical Society

Multisite Phosphorylation Modulates the T Cell Receptor ζ-Chain Potency but not the Switchlike Response

Himadri Mukhopadhyay,[1] Ben de Wet,[1] Lara Clemens,[3] Philip K. Maini,[2] Jun Allard,[3] P. Anton van der Merwe,[1,*] and Omer Dushek[1,2,*]
[1]Sir William Dunn School of Pathology and [2]Wolfson Centre for Mathematical Biology, Mathematical Institute, University of Oxford, Oxfordshire, United Kingdom; and [3]Department of Mathematics, University of California-Irvine, Irvine, California

ABSTRACT Multisite phosphorylation is ubiquitous in cellular signaling and is thought to provide signaling proteins with additional regulatory mechanisms. Indeed, mathematical models have revealed a large number of mechanisms by which multisite phosphorylation can produce switchlike responses. The T cell antigen receptor (TCR) is a multisubunit receptor on the surface of T cells that is a prototypical multisite substrate as it contains 20 sites that are distributed on 10 conserved immunoreceptor tyrosine-based activation motifs (ITAMs). The TCR ζ-chain is a homodimer subunit that contains six ITAMs (12 sites) and exhibits a number of properties that are predicted to be sufficient for a switchlike response. We have used cellular reconstitution to systematically study multisite phosphorylation of the TCR ζ-chain. We find that multisite phosphorylation proceeds by a nonsequential random mechanism, and find no evidence that multiple ITAMs modulate a switchlike response but do find that they alter receptor potency and maximum phosphorylation. Modulation of receptor potency can be explained by a reduction in molecular entropy of the disordered ζ-chain upon phosphorylation. We further find that the tyrosine kinase ZAP-70 increases receptor potency but does not modulate the switchlike response. In contrast to other multisite proteins, where phosphorylations act in strong concert to modulate protein function, we suggest that the multiple ITAMs on the TCR function mainly to amplify subsequent signaling.

INTRODUCTION

Protein phosphorylation is a ubiquitous mechanism of signal transduction that is regulated by the opposing actions of kinases and phosphatases (1,2). Failure to regulate phosphorylation can lead to a number of abnormal cellular phenotypes, such as constitutive cell growth, which is the case in many cancers (1,3). The majority of eukarotyic proteins are phosphorylated on more than one site (4,5), raising the possibility that multisite phosphorylation may confer an additional regulatory mechanism (6,7).

A well-known regulatory mechanism is a switchlike response, whereby the phosphorylation state of a protein can be highly sensitive to the concentrations of its modifying kinase and phosphatase. A large body of work has focused on understanding whether multiple phosphorylation sites on a single substrate can confer switchlike responses. Mathematical models have shown that switchlike responses can be produced by a number of mechanisms that rely on multiple phosphorylation sites, such as substrate sequestration (8), nonessential sites (9), local concentrations (10), independent binding sites (11), membrane-anchoring (diffusion-limited reactions) (12), entropic mechanisms (13), binding of effector molecules (14), cooperativity between sites, or site-specific enzymatic rates (15), and other mechanisms reviewed elsewhere (2,6,7). The key difference between the models lies in the assumptions about how the sites are modified. For example, a switchlike response can be produced when the modifying enzymes phosphorylate the substrate in a precise sequence but not when modification is random (Fig. S1 in the Supporting Material). Despite extensive mathematical studies, there are few experimental studies aimed at a systematic investigation of multisite phosphorylation.

The T cell antigen receptor (TCR) on the surface of T cells is a prototypical multisite substrate (16). T cells

Submitted December 11, 2015, and accepted for publication March 15, 2016.

*Correspondence: omer.dushek@path.ox.ac.uk or anton.vandermerwe@path.ox.ac.uk
Himadri Mukhopadhyay and Ben de Wet contributed equally to this work.

Editor: H. Wiley.
http://dx.doi.org/10.1016/j.bpj.2016.03.024

© 2016 Biophysical Society
This is an open access article under the CC BY license (http://creativecommons.org/licenses/by/4.0/).

CrossMark

rely on the TCR to recognize antigens, in the form of peptides bound to major histocompatibility complexes, on the surfaces of antigen-presenting cells. Ligation of the TCR by peptides bound to major histocompatibility complexes can trigger a large signal transduction cascade in T cells that may lead to a number of functional responses, such as proliferation, differentiation, and the secretion of effector cytokines (17,18). The T cell response is of crucial importance in the elimination of infections and pathologies, and the ability to harness this response is a central objective in immune-based therapies (19).

The TCR is a multisubunit receptor that contains two ligand binding chains that associate with six signaling chains that collectively contain 20 phosphorylation sites. These sites are distributed on 10 immunotyrosine-activated-based motifs (ITAMs) with consensus sequence YXX[L/I]$X_{6–9}$YXX[L/I], where X is any amino acid (20). While four of the signaling chains each contain one ITAM, the T cell receptor ζ-chains, which form a homodimer by a disulfide bond in the transmembrane domain, each contain three ITAMs (or six ITAMs across the dimer). These sites are phosphorylated by the membrane-anchored tyrosine kinase Lck and Fyn and dephosphorylated by the transmembrane phosphatases CD148 and CD45. When both tyrosines in an ITAM are phosphorylated they generate docking sites for the tandem SH2 domains of the cytosolic tyrosine kinase ZAP-70. Bound ZAP-70 can phosphorylate tyrosines on other substrates that initiate the signal transduction that may lead to T cell activation (17).

Given that both kinases and phosphatases are constitutively active in resting T cells (16,21), it is thought that the phosphorylation state of the TCR is tightly regulated. This is underlined by the observation that even a single TCR ligand is sufficient to activate T cells (22).

A number of the multisite phosphorylation mechanisms that produce switchlike responses discussed above may be applicable to the T cell receptor ζ-chain. The ζ-chain has been shown to be sequentially phosphorylated (23,24), which is sufficient to produce switchlike responses (Fig. S1). Moreover, it contains nonessential sites because even a single phosphorylated ITAM is sufficient to recruit and activate ZAP-70, it has the potential to be sequestered in the membrane when fully dephosphorylated (25), and components of the signaling module are membrane-anchored. We have also used a mathematical model to show that differential binding affinities of ZAP-70 to each of the ζ-chain ITAMs combined with sequential phosphorylation can produce switchlike responses (14).

In this work, we have investigated how the phosphorylation state of the ζ-chain is regulated by changes to the kinase/phosphatase ratio as the number of phosphorylation sites is modified. To do this, we performed cellular reconstitution of 21 distinct signaling modules that include different combinations of wild-type or mutant forms of the TCR ζ-chain, Lck, CD148, and ZAP-70. By reconstituting ζ-chains with all combinations of ITAM mutants, we found no evidence for sequential phosphorylation, and surprisingly, we found that phosphorylation sites did not modulate the switchlike response but did modulate receptor potency and maximum phosphorylation. We show that a phosphorylation-dependent enhancement in the binding rate of the enzymes can explain these observations and that a molecular mechanism that is consistent with such an enhancement is a disorder-to-order transition of the ζ-chain upon phosphorylation.

MATERIALS AND METHODS

Plasmids

All sequences used in this study were of murine origin unless otherwise stated. TCR ζ-chain variants were generated as previously described in Choudhuri et al. (26). Briefly, the transmembrane and cytoplasmic domains of mouse TCR ζ-chain were cloned in frame with the extracellular domains of rat CD2 in the mammalian expression vector pcDNA3.1(+). For each ITAM mutant, both ITAM tyrosines were mutated to phenylalanine. Lck was cloned into the mammalian expression vector pEF3 as a constitutively active Y505F-variant (27). An SH2-binding mutant, Lck(R154K), was generated by QuikChange PCR (Agilent Technologies, Santa Clara, CA). Ectodomain-truncated CD148, encoding the last two C-terminal fibronectin type III, transmembrane, and catalytic domains, as well as an extracellular FLAG-tag, was generated as previously described in Cordoba et al. (28), and cloned in the expression vector pcDNA3.1(+). ZAP-70 was cloned into a pcDNA3.1(+) vector, and the low activity variant (ZAP-70*) containing the SH2-binding mutations, R41K and R190K, as well as the catalytically dead mutation, K368A, was generated by QuikChange PCR (Agilent Technologies).

Cell culture and transfection

HEK 293T cells were cultured at 37°C and in a 10% CO_2 in DMEM containing 10% fetal bovine serum. A day before transfection, cells were seeded to reach 70% confluence, and a single 175 cm^2 flask was transiently transfected for each experimental condition. For experiments including only Lck, CD148, and TCR ζ-chain variants, 8 μg of each plasmid (24 μg total) was complexed with 24 μg of branched-chain PEI (polyethylenimine, average M_w 25,000; Sigma-Aldrich, St. Louis, MO) in 0.9 mL serum-free medium for 10 min, before dropwise addition to cells in serum-free medium. An additional 25 μg of ZAP-70 DNA and 25 μg of PEI was included in transfection mixtures in experiments containing ZAP70. Cells were incubated at 37°C for 4 h before transfection medium was replaced with complete medium supplemented with fetal bovine serum. Cells were harvested after 24 h by gentle shaking, centrifuged, and resuspended in fresh serum-free medium at an appropriate density for assay.

Pervanadate treatment of transfectants

Basal kinase/phosphatase balance was disrupted by inhibition of phosphatase activity with a serial dilution of pervanadate. Pervanadate was generated immediately before the assay by incubating 10 mM sodium orthovanadate with 0.7% H_2O_2 at room temperature for 10 min. H_2O_2 was inactivated by the addition of 20 μg/mL catalase at room temperature for 10 min. A 24-step, 1.25-fold serial dilution of pervanadate was made up in serum-free medium starting at 1 mM. Harvested transfectants were plated at 300,000 cells per well in 96-well assay plates in a volume of 60 μL/well and treated with 60 μL/well pervanadate serial dilutions for

30 min at 37°C in a tissue culture incubator. Reactions were terminated and cells were lysed by adding 120 μL/well lysis buffer containing 2% Nonidet P-40 substitute (Roche, Indianapolis, IN), Mammalian Protease Inhibitor Cocktail (Sigma-Aldrich), and 250 μM sodium orthovanadate in TBS (20 mM Tris, pH 7.5, 150 mM NaCl). Cellular debris was removed by centrifugation and lysates were transferred to wells of previously prepared immunosorbent capture plates.

Immunosorbent assay of TCR ζ-chain phosphorylation

TCR ζ-chain phosphorylation was quantified in immunosorbent assay by capturing chimeric TCR ζ-chain with anti-rat CD2 antibody and detecting phosphorylation with anti-phosphotyrosine antibody. A day before assay, MaxiSorp flat-bottom 96-well plates (Nalge Nunc, Rochester, NY) were coated overnight at 4°C with 100 μL/well anti-rat CD2 antibody (AbD Serotec) at 0.7 μg/mL in carbonate-bicarbonate buffer, pH 9.6. On the day of the assay, capture antibody was decanted and wells were blocked with 200 μL 1% bovine serum albumin in TBS for 1 h at room temperature. Plates were washed three times with TBS-T buffer (TBS containing 0.05% TWEEN-20). Cleared saturating amounts of lysate was transferred to plates and TCR ζ-chain captured for 2 h at 4°C. Lysate was decanted and plates were washed 6 times with TBS-T. Phosphorylation was probed by addition of 100 μL/well of biotinylated anti-phosphotyrosine antibody (clone pY20; BioLegend, San Diego, CA) at 1 μg/mL, and plates were incubated for 1 h at room temperature. Plates were subsequently washed 3 times with TBS-T. Biotinylated antibody was probed with 100 μL/well of 0.1 μg/mL IRDye 800CW-streptavidin (LI-COR Biosciences, Lincoln, NE) for 1 h at room temperature. Plates were washed three times with TBS-T, dried, and fluorescence-detected by scanning plates in an Odyssey Sa Infrared Imaging System (LI-COR).

Flow cytometry

Transfectants were blocked by incubating 1×10^6 cells for 15 min on ice in 100 μL 1% PBS-A (albumin in phosphate-buffered saline). Surface antigens were stained by incubating for 1 h on ice with 1 μg/ml FITC-conjugated anti-FLAG antibody (Sigma-Aldrich) and 1 μg/mL phycoerythrin-conjugated anti-rat CD2 (AbD Serotec, Kidlington, Oxfordshire, UK) in 100 μL PBS-A. Cells were washed three times in PBS and fixed with 4% paraformaldehyde in PBS for 15 min at room temperature. Cells were washed three times in PBS and permeabilized in 0.1% saponin (Sigma-Aldrich) in PBS-A. Intracellular antigens were stained by incubating for 1 h at room temperature with 1 μg/mL Alexa Fluor 647-conjugated anti-mouse Lck (BD Biosciences, San Jose, CA) and 1 μg/mL Pacific Blue-conjugated anti-mouse ZAP-70 (Thermo Fisher Scientific, Guilford, CT) in 100 μL PBS-A. Cells were washed three times with PBS and analyzed using a FACScan flow cytometer (Becton Dickinson, Franklin Lakes, NJ).

Curve fitting, normalization, and statistical analyses

The steady-state phosphorylation profiles were fit to a logarithmic Hill function in Prism (GraphPad Software, La Jolla, CA), that produces estimates of E_{min} and E_{max}, which correspond to the minimum and maximum values, respectively, $\log_{10}(EC_{50})$, which is the logarithmic value of the kinase/phosphatase that yields half of the maximal response (otherwise known as potency), and n, which is the Hill number that determines the degree of the switchlike response (also known as the sensitivity).

We used an index module (wild-type ζ 123) as our standard curve so that experimental data can be averaged across different days. This normalization was performed by (1) dividing the response for each module by the value of E_{max} for the index module and (2) by subtracting the pervanadate concentration series on a log-scale (x axis) by the value of EC_{50} for the index module. Fitted Hill numbers were normalized to the value of the index module.

All statistical analyses were carried out on normalized averaged quantities using a t-test in Prism (GraphPad Software). Given that the Hill number and $\log_{10}(EC_{50})$ of the index module was 1 and 0, respectively, we used a t-test to determine whether the corresponding values for the group of single ITAM mutants (ζX23, ζ1X3, and ζ12X) or the double ITAM mutants (ζ1XX, ζX2X, and ζXX3) differed from these values. An unpaired t-test was used to compare between the group of single and double ITAM mutants.

RESULTS

Cellular reconstitution of multisite phosphorylation of the T cell receptor ζ-chain

To study the effects of multiple phosphorylation sites on the T cell receptor ζ-chain, we reconstituted four components in various combinations (Fig. 1 *A*). The wild-type substrate was a chimera consisting of the ζ-chain transmembrane and cytosolic domains but with the extracellular domain of rat CD2 (previously used as a simplified receptor (26)). The wild-type kinase was Lck with a point mutation to a negative regulatory tyrosine (Y505F) that renders the kinase constitutively active. The wild-type phosphatase was CD148 with a truncated extracellular domain, which was previously shown to have higher expression (28). Lastly, we have used wild-type ZAP-70 in a number of experiments.

Combinations of the wild-type components or mutant variants were transfected into HEK293 cells (Fig. 1 *B*), which comprise a nonhematopoietic human cell line that is not expected to express any of the endogenous T cell receptor proximal signaling proteins (29). We found that expression peaked 24 h posttransfection (not shown), and therefore all experiments were performed at this time point. Using a k-means clustering algorithm, we found that cells were readily classified into two distinct populations that were either positive for all three components or were negative for all three components (Fig. 1 *B*). Moreover, component expression in the positive population was correlated (Fig. S2), which has been previously shown to be the case in transfections of HEK293 cells (30).

To determine the total phosphorylation profile of the ζ-chain, the cells were treated with a titration of the tyrosine phosphatase inhibitor pervanadate for 30 min before the ζ-chain was captured using an anti-CD2 antibody and phosphorylation detected using an anti-phosphotyrosine antibody (Fig. 1 *C*). We found limited phosphorylation without pervanadate treatment and moreover, treatment with the Lck inhibitor PP2 did not lead to detectable reduction in phosphorylation (not shown), suggesting that CD148 dominates over Lck basally. Given that HEK293 cells are expected to have endogenous tyrosine kinases and phosphatases, we determined their contribution by transfecting in the substrate with only Lck or only CD148. Although we observe dephosphorylation in the absence of exogenous

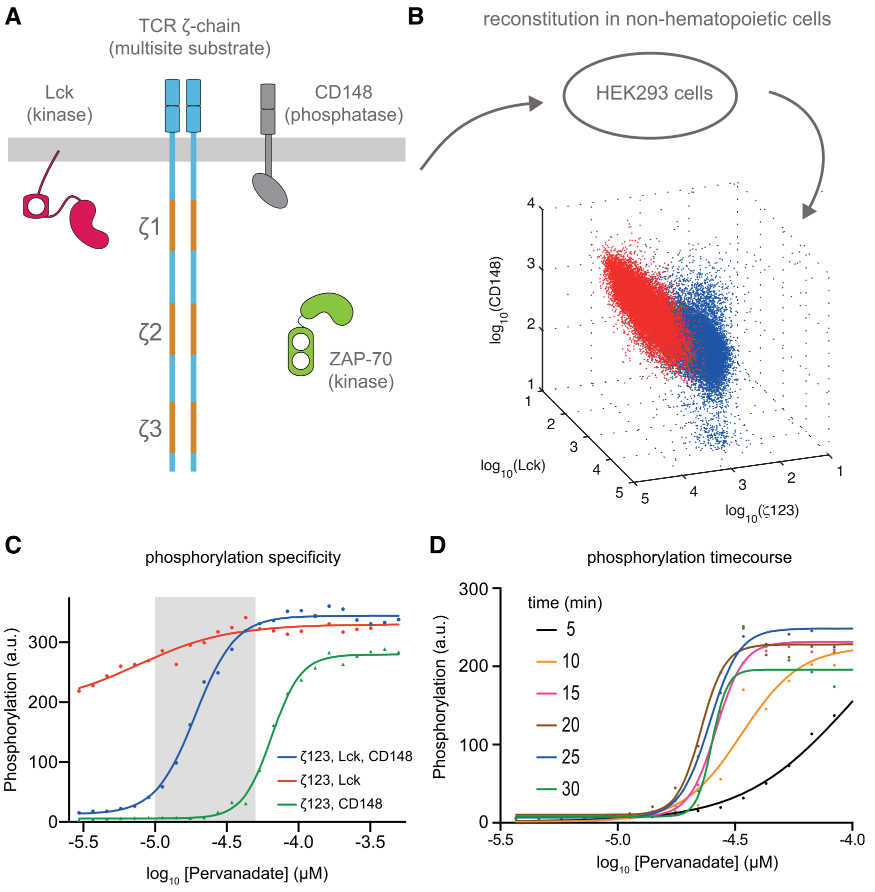

FIGURE 1 Cellular reconstitution of multisite phosphorylation of the T cell receptor ζ-chain. (*A*) Schematic of reconstituted signaling proteins. The substrate is a CD2-TCRζ-chain chimera that contains six phosphorylation sites distributed on three ITAMs (*orange*) that dimerizes as a result of a disulfide bond in the ζ-chain transmembrane domain (i.e., the substrate is a receptor dimer that contains 12 phosphorylation sites). The substrate is phosphorylated by the membrane-anchored kinase Lck and dephosphorylated by the transmembrane phosphatase CD148. The cytosolic kinase ZAP-70 can bind to phosphorylated ITAMs by SH2 domains. (*B*) Combinations of these components were transfected into the nonhematopoietic HEK293 cell line and molecular expression was detected with flow cytometry 24 h posttransfection. A *k*-means clustering algorithm classified the cells as either positive (*red*, 25%) or negative (*blue*, 75%) for transfected components (see Fig. S2 for two-dimensional projections). (*C*) Cells transfected with the indicated components were incubated with increasing concentrations of the tyrosine phosphatase inhibitor pervanadate for 30 min (*x* axis) before total phosphorylation of the ζ-chain was determined (*shaded rectangle* highlights the specificity range). (*D*) Phosphorylation time course for reconstitution of Lck, CD148, and ζ-chain indicates that steady-state phosphorylation is achieved by ~15–20 min. See Materials and Methods for experimental details. To see this figure in color, go online.

CD148 or phosphorylation in the absence of exogenous Lck, a large range exists where phosphorylation is regulated primarily by exogenous Lck and CD148 (*shaded region*, Fig. 1 *C*). Moreover, a timecourse revealed that by ~15–20 min, the system has come to steady state (Fig. 1 *D*).

Multisite phosphorylation of the T cell receptor ζ-chain modulates potency but not the switchlike response

To determine the effects of multiple phosphorylation sites, we reconstituted signaling modules where the number of ζ-chain ITAMs is modified. We reconstituted Lck, CD148, and either the wild-type TCR ζ-chain, ζ-chain with one ITAM mutated (ζX23, ζ1X3, and ζ12X), or ζ-chain with two ITAMs mutated (ζ1XX, ζX2X, and ζXX3), where *X* indicates that the two ITAM tyrosines have been mutated to phenylalanine (Fig. 2 *A*).

As expected, a reduced ITAM number led to a reduced maximum phosphorylation by ~1/3 per mutated ITAM. The observation that the maximum phosphorylation depends on the number of ITAMs, but not their identity, suggests that a strict sequential mechanism of phosphorylation is unlikely to be operating. Rather, it is more likely that ζ-chain phosphorylation follows an unstructured random modification mechanism.

To determine the effects of multisite phosphorylation on potency and the switchlike response, we fit a Hill function to the phosphorylation profile to determine EC_{50} (potency) and the Hill number (switchlike response). We found no significant difference in the Hill number between the wild-type ζ-chain, which as a dimer contains 12 phosphorylation sites, and ζ-chains with ITAM mutations (Fig. 2 *B*). This observation is also consistent with a random mechanism of phosphorylation because, as discussed in the introduction, a sequential mechanism predicts that the switchlike response will be modulated by the number of sites (Fig. S1).

While the advantage of the cellular reconstitution system used here is that it maintains a normal cellular environment, a key drawback is that chemical inhibitors must be used to perturb the kinase-to-phosphatase balance. We have observed absolute Hill numbers near 4 but we are unable to rule out the possibility that these large Hill numbers were a result of a nonlinear relationship between the concentration of inhibitor and the concentration of active phosphatase. Therefore, we confined our analysis to changes in the Hill number (and other parameters) by reporting normalized parameter values as the number of phosphorylation sites is modified (see Materials and Methods).

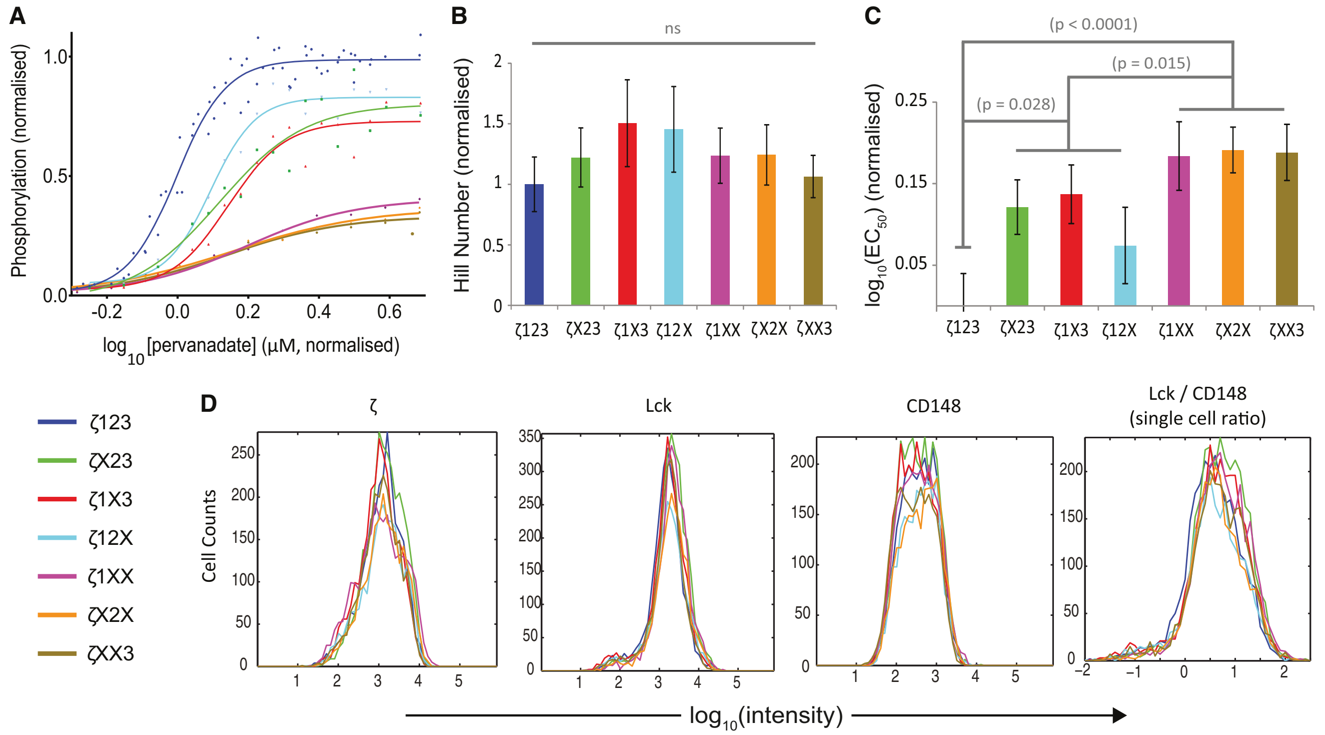

FIGURE 2 Multisite phosphorylation of the T cell receptor ζ-chain enhances potency but not the switchlike response. (*A*) Phosphorylation profiles of reconstituted signaling modules containing Lck, CD148, and either wild-type ζ-chain containing all three ITAMs (ζ123) or all possible combination of ITAM mutations that replace the two ITAM tyrosines with phenylalanine. A Hill function is fit to all curves to produce estimates of the (*B*) the Hill number and (*C*) potency (EC_{50}). (*D*) Expression of ζ-chain, Lck, CD148, and the ratio of Lck to CD148 at the single cell level is comparable for the seven reconstituted signaling modules (see Fig. S4 for comparison of mean expression and percent positive). Representative data (*A* and *D*) and averaged parameters (*B* and *C*) are normalized to the index module (Lck, CD148, and ζ123) with error bars indicating mean ± SE. See Materials and Methods for details on normalization and statistical analysis. To see this figure in color, go online.

We observed a positive correlation between receptor potency and the number of sites (Fig. 2 *C*). This effect is not predicted by the standard models of multisite phosphorylation where potency often remains unchanged as the number of sites changes (Fig. S1). A simple (and trivial) explanation for the modification in potency is that Lck and/or CD148 have different binding affinities or catalytic rates for each ITAM. However, this explanation can be ruled out by the observation that the phosphorylation profile of the three ζ-chain constructs with only a single intact ITAM (ζ1XX, ζX2X, and ζXX3) is similar, implying that both enzymes are equally efficient at modifying each of the three ITAMs. We conclude that it is the juxtaposition of multiple phosphorylation sites (or ITAMs) in the wild-type ζ-chain configuration that elicits a cooperative effect that increases potency.

To rule out that the differences in the phosphorylation profiles are a result of differences in molecular expression, we performed flow cytometry for every reconstituted signaling module. We found that the percent of cells positive for the three components and the expression levels of the three components in the seven reconstituted modules were similar (Figs. 2 *D* and S4). Moreover, we did not find any significant correlations between expression level and parameters extracted from the phosphorylation profiles (not shown). We also found that the phosphorylation kinetics is similar for ITAM mutants (Fig. S3).

Given that Lck contains an SH2 domain that can interact directly with phosphorylated sites on the ζ-chain, we wondered whether this domain could explain the observed changes in potency. This recruitment could increase the local concentration of Lck in the vicinity of the ζ-chain leading to enhanced efficiency of phosphorylation, which would be greatest for wild-type ζ that contains the largest number of sites. We introduced a point mutation to the Lck SH2 domain that is known to abolish binding (Lck-R154K) and performed the reconstitution experiments on all seven ζ-chain mutants. We found that the observed changes in potency persisted, suggesting that the SH2 domain of Lck is not responsible for increased potency (Fig. S5).

ZAP-70 modulates T cell receptor ζ-chain potency but not the switchlike response

We have previously used a mathematical model to show that ZAP-70 can produce increases in both potency and the switchlike response of the T cell receptor ζ-chain (14). The predicted increase in the switchlike response was critically dependent on sequential phosphorylation and on

ZAP-70 binding to the three ζ-chain ITAMs with different affinities, as previously reported in Love and Hayes (20) and Isakov et al. (31). We therefore assessed the effects of ZAP-70 on multisite ζ-chain phosphorylation.

We first compared the effects of ZAP-70 on the wild-type ζ-chain by reconstituting Lck, CD148, and ζ-chain with either wild-type ZAP-70 or a low activity ZAP-70 with inactivating point mutations to the SH2 and catalytic domain(s) (ZAP-70*). These two domains are known to favor phosphorylation; the SH2 domains can bind with high affinity to phosphorylated ITAMs protecting them from dephosphorylation and the catalytic domain may be important to directly phosphorylate ITAM tyrosines. We compare ZAP-70 with ZAP-70* to control for the total protein load imposed on the HEK293 cells by reconstitution.

Comparing the phosphorylation profiles of ZAP-70 and ZAP-70* revealed the expected increase in potency but no change to the switchlike response was observed (Fig. 3, *A–C*), which is consistent with a recent report from Hui and Vale (32). This result is also consistent with our previous theoretical prediction that ZAP-70 will not enhance the switchlike response if the ζ-chain is modified by a nonsequential random mechanism (14).

We next examined the contribution of each ITAM to the ZAP-70-mediated increase in potency observed for the wild-type ζ-chain. We reconstituted Lck, CD148, and ZAP-70, with all ζ-chain variants (Fig. 3, *D–F*) and observed, as before, no trend in the Hill number but a significant increase in potency with the number of phosphorylation sites. Importantly, we did not observe any differences in potency within the ζ-chain group that contained two ITAMs or within the ζ-chain group that contained one ITAM, suggesting that ZAP-70 binds all ζ-chain ITAMs in vivo with similar affinities. As before, we examined the expression of all components to ensure that any differences in the phosphorylation profiles were not a result of differences in molecular expression (Figs. S6 and S7).

In summary, ZAP-70 enhances the phosphorylation potency of the TCR ζ-chain but does not alter the switchlike response. Moreover, the correlation between the potency and the number of phosphorylation sites observed in the absence of ZAP-70 is maintained in its presence.

Phosphorylation-dependent binding rates are sufficient to explain the modulation of potency by multisite phosphorylation

We have found a correlation between the number of phosphorylation sites or ITAMs with receptor potency that cannot be explained by standard multisite phosphorylation models (see Discussion). Moreover, the observation that the group of three single (ζX) and three double (ζXX)

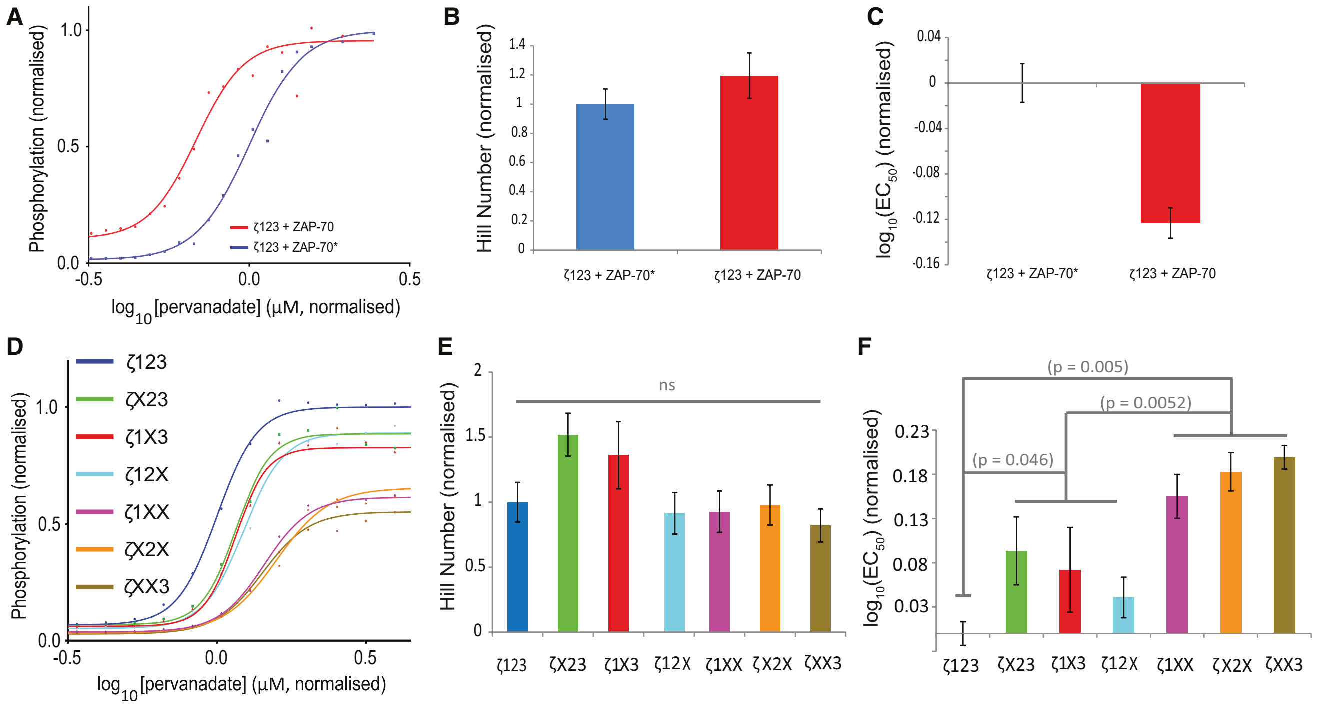

FIGURE 3 ZAP-70 enhances potency but not the switchlike response of ζ-chain phosphorylation. (*A–C*) Phosphorylation profiles of reconstituted signaling modules containing Lck, CD148, ζ-chain, and either wild-type ZAP-70 or ZAP-70* that contains point mutations to abolish SH2 domain binding and tyrosine kinase function. (*D–F*) Phosphorylation profiles of reconstituted signaling modules containing Lck, CD148, ZAP-70, and either wild-type ζ-chain or all possible combinations of ITAM mutations. Component expression is comparable in all signaling modules (Figs. S6 and S7). Representative data (*A* and *D*) and averaged parameters (*B*, *C*, *E*, and *F*) are normalized to an index module (Lck, CD148, ζ123, and ZAP-70) with error bars indicating mean ± SE. See Materials and Methods for statistical analysis. To see this figure in color, go online.

ITAM mutant ζ-chains exhibited similar profiles indicates that Lck and CD148 modify each ITAM with similar efficiencies ruling out a trivial explanation for the enhanced potency. We therefore hypothesized that the number of phosphorylated ITAMs, independent of their specific identity, determines the rate of enzymatic modification.

We formulated a mathematical model that includes the six ITAMs on the ζ-chain dimer (Fig. 4 *A*). Because we do not have data relating to the effect of individual tyrosines in an ITAM we have coarse-grained the two tyrosines of each ITAM into an effective single site. We model a phosphorylation-dependent enhancement (or priming) of enzymatic efficiencies by enhancing k_{on} for both enzymes with increasing levels of ζ-chain phosphorylation. To do so, we introduce a new parameter, λ, which is the fold increase in the on-rate per ITAM phosphorylation so that after all ITAMs are phosphorylated the on-rate is increased by $\lambda_{max} = \lambda^6$. Because all calculations are in the steady state, we found similar results if enhancement is applied to k_{off} or k_{cat} (not shown).

Computations with the mathematical model for $\lambda = 3$ (or $\lambda_{max} = 729$) produced changes in potency without large differences in the Hill number (Fig. 4 *B*). The value of λ that can reproduce these results was not unique because a parameter scan revealed a region of $\lambda - k_{on}$ space that is consistent with changes in potency without changes in the Hill number (Fig. S8). We found that the changes in potency can be reproduced with a sufficiently large k_{on} when $\lambda = 1$, but in this limit there was residual phosphorylation that we did not observe in our experimental data (Fig. S8 *A*). Therefore, we conclude that a phosphorylation-dependent enhancement is the most likely explanation for the correlation between the number of ITAMs and receptor potency.

We next sought a plausible molecular mechanism that can be responsible for the phosphorylation-dependent enhancement. It has been previously proposed that molecular entropy may be reduced by phosphorylation of disordered amino-acid chains (33). In this model, the flexible disordered amino-acid polymer can adopt many configurations when free, but the binding of a catalytic domain reduces the number of configurations and therefore an entropic penalty to binding is incurred. This penalty can be substantially reduced if phosphorylation decreases the number of configurations of the free polymer.

Given that the dimeric ζ-chain is predicted to be disordered based on the sequence of its cytoplasmic chain (determined using established predictions algorithms (34,35)), we used a polymer model to determine an upper bound on the maximum fold-increase in the on-rate (λ_{max}). The model includes the ζ-chain dimer anchored to the plasma membrane with each polymer in the dimer, modeled as a freely jointed chain with variable number of segments but a fixed overall length. The length of each segment is known as the Kuhn length (36). To calculate an upper bound for λ_{max}, we compare the probability that the catalytic domain of an enzyme can bind the fully dephosphorylated ζ-chain (maximum disorder) to a hypothetical situation where the ζ-chain is completely ordered (i.e., exists in a single state where there is no entropic penalty to bind) as a result of maximum phosphorylation (Fig. 5 *A*).

There are two factors that determine λ_{max} in the model: the catalytic domain size and the Kuhn length. Larger

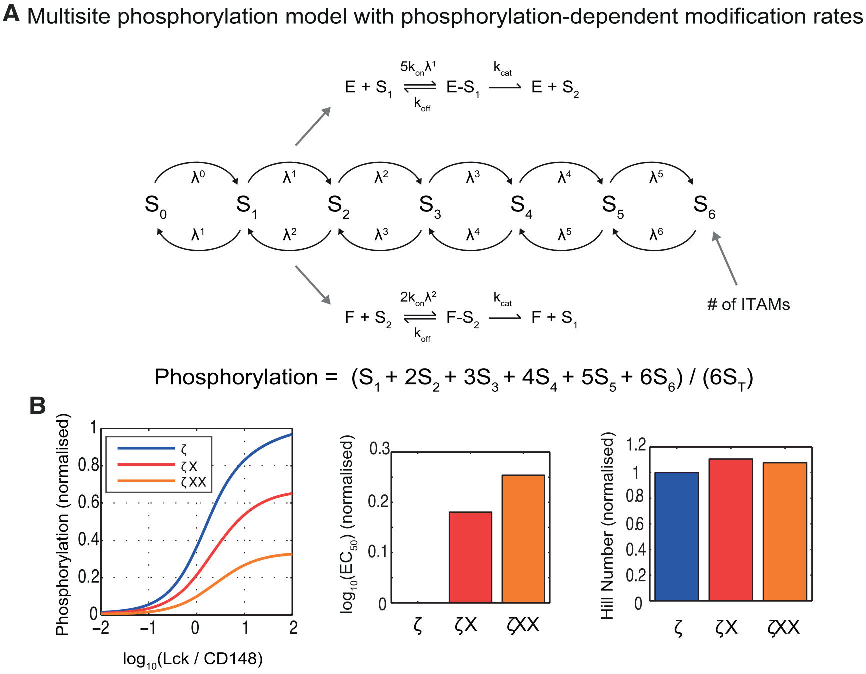

FIGURE 4 A phosphorylation-dependent enhancement in enzymatic efficiencies is sufficient to explain experimental results. (*A*) Multisite phosphorylation model showing the transitions between the concentration of phosphorylated substrate on the indicated number of ITAMs that is mediated by the kinase (*E*) and the phosphatase (shown as *F*). The model includes a fold-enhancement in the on-rate (λ) that is proportional to the number of phosphorylated ITAMs with the maximum increase $\lambda_{max} \sim \lambda^6$. (*B*) Phosphorylation profiles for a 6-ITAM (ζ), 4-ITAM (ζX), and a 2-ITAM (ζXX) substrate calculated using $\lambda = 3$ ($\lambda_{max} = 729$) showing the potency and Hill numbers. See the Supporting Material for computational details. To see this figure in color, go online.

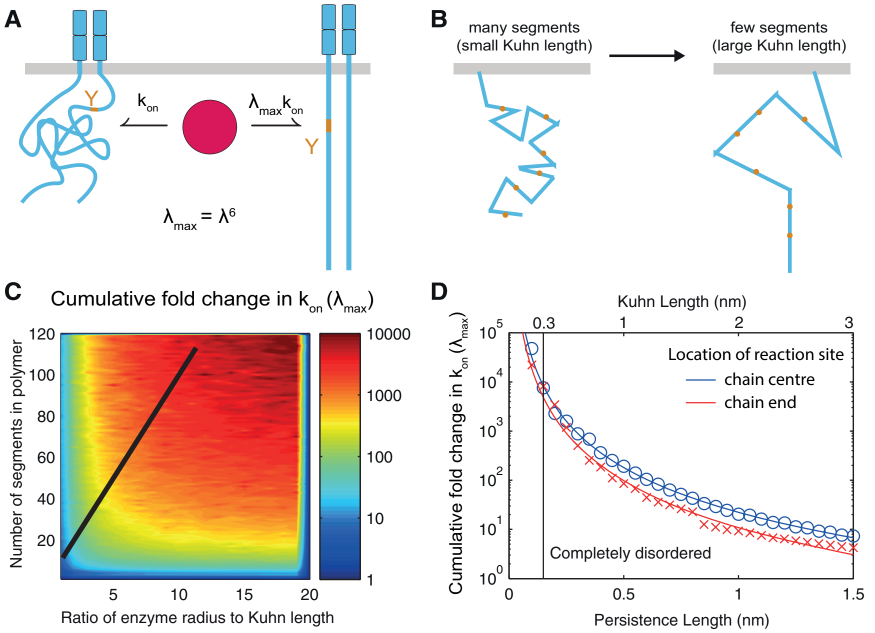

FIGURE 5 A phosphorylation-dependent enhancement in the on-rate can arise from a disorder-to-order transition. (*A*) A polymer model of the ζ-chain predicts that binding of the kinase (or phosphatase) is impeded by entropic disorder (*left*) and, if phosphorylation imposes local order, this impedance will be reduced when the substrate is phosphorylated with the maximal reduction occurring when the substrate is fully phosphorylated (*right*). Larger catalytic domains will incur a larger entropic penalty of binding. (*B*) The degree of disorder is determined by the number of segments whose length is known as the Kuhn length. (*C*) Heat map showing λ_{max} for different segment numbers (*y* axis) and for different ratios of the catalytic domain radius to Kuhn length (*x* axis). (*D*) Comparison of maximal enhancement over the Kuhn length (or persistence length) when the enzymatic binding site is at the center of the ζ-chain (*blue*) or at the membrane-distal tyrosine at position 152 (*red*). A Kuhn length of ~0.5 or ~2 amino acids corresponds to λ_{max} ~ 700. Black line in (*C*) corresponds to parameter range in (*D*). See Materials and Methods for computational details. To see this figure in color, go online.

catalytic domains will incur a larger entropic penalty (increasing λ_{max}) and smaller Kuhn lengths increase the basal number of configurations (increasing λ_{max}) (Fig. 5, *A* and *B*). We therefore generated a heat map showing λ_{max} when these parameters are varied (Fig. 5 *C*) and also show a specific slice of the heat map for clarity (Fig. 5 *D*). When the Kuhn length is 0.3 nm (or approximately the length of the C_α–C_α bond), we find that $\lambda_{max} \approx 8000$; whereas $\lambda_{max} \approx 700$ (used in the phosphorylation model above) can be found when the Kuhn length is 0.5 nm, which is consistent with the flexibility previously reported for polypeptide chains (37).

These calculations are based on the enzyme catalytic domain interacting with a test tyrosine at the center of the chain, but we have also performed the calculations with a test tyrosine at position 152, which is the most distal tyrosine for murine ζ-chain, finding only a weak dependence on λ_{max} (Fig. 5 *D*).

In summary, a phosphorylation-dependent enhancement in the binding interactions can produce the observed correlation between the number of phosphorylation sites and receptor potency, and this enhancement is consistent with a disorder-to-order transition upon phosphorylation that reduces the molecular entropic penalty to binding.

DISCUSSION

Using cellular reconstitution, we have performed systematic experiments to test predictions of mathematical models for multisite phosphorylation as applied to the T cell receptor ζ-chain. In contrast to model predictions, we have found no evidence that multiple phosphorylation sites are able to modulate the switchlike response but have found that multiple sites can modulate receptor potency and maximum phosphorylation.

Reconstitution is a powerful reductionist method that enables the study of a well-defined signaling module without the feedbacks and unknown interactions that occur in native cells. Hui and Vale (32) reconstituted the TCR ζ-chain with different concentrations of Lck and CD45 on liposomes using purified proteins and found only a modest Hill number of 2 that did not change when ZAP-70 was included, which is consistent with our finding. James and Vale (30) reconstituted the TCR and a number of other TCR proximal components in HEK293 cells to investigate the mechanism by which ligand binding to the TCR induces intracellular phosphorylation, a process termed “TCR triggering” (16). In contrast to their work, we did not find detectable basal phosphorylation when reconstituting the TCR, Lck, and CD148 alone in HEK293 cells. This discrepancy is likely a result of the high expression of our extracellular domain truncated phosphatase (38).

This work has several implications for TCR proximal signaling. We have previously used a mathematical model to show that multiple ζ-chain ITAMs, their sequential phosphorylation, and differences in ZAP-70 binding affinities can generate emergent switchlike responses (14). However, we found no evidence for sequential phosphorylation of the TCR ζ-chain in this work. Two previous studies have provided evidence for sequential phosphorylation but differed in the reported sequence (23,24). The discrepancy with this work may be explained by the fact that the previous studies relied on measurements without systematic variations to kinase/phosphatase. Moreover, we found no evidence for in vivo differences in ZAP-70 binding affinities in this work because, for example, the potencies of ζ-chains

with single intact ITAMs were identical in the presence of ZAP-70 (Fig. 3 *F*). Previous work has suggested differences in ZAP-70 binding affinities ranging from 3- to 30-fold based on measurements with the isolated tandem SH2 domains (31,39) but a more recent study has shown markedly different behavior of the full protein (40). We have found that ZAP-70 increases receptor potency but not the switchlike response consistent with a previous study by Hui and Vale (32). The combination of random phosphorylation and similar ZAP-70 binding affinities predicts that multiple ITAMs will not contribute to a switchlike response (see Fig. 5A in Mukhopadhyay et al. (14)), which is the observation made in this work.

What is then the purpose of multiple ITAMs in the TCR complex? Although the TCR ζ-chain ITAMs are not sequentially phosphorylated, they may be involved in kinetic proofreading, which relies on a delay (that can be produced by either distributive sequential or distributive random phosphorylation) between initial ligand binding and downstream signaling for antigen discrimination (18,20,41–46). The effect with the largest magnitude is the intuitive difference in the maximum phosphorylation when ITAMs are removed. This likely reflects the signal amplification property of the TCR and is consistent with previous work showing that, for example, T cell proliferation scales with the number, but not the identity, of individual ITAMs (47) and more recent work showing that the ζ-chain is dispensable for many T cell responses (48). This leaves open the question of why there are highly conserved differences in individual ITAMs within the TCR (see, for example, Fig. S1 in Mukhopadhyay et al. (14) for an alignment).

The observation that phosphorylation of one ITAM can enhance the phosphorylation of other ITAMs is related to the phenomenon termed “phosphorylation priming”. Phosphoproteomic analysis revealed that phosphorylation of one site can enhance the phosphorylation of neighboring sites within ~10 amino acids (49). Moreover, recent studies have found evidence for such phosphorylation priming in an actin cross-linking protein (50) and within the two tyrosines of the ITAM (51).

The mechanism underlying phosphorylation priming is incompletely understood. A possible mechanism is a disorder-to-order transition that phosphorylation may induce in intrinsically disordered regions of proteins (33). The T cell receptor ζ-chain, and indeed many noncatalytic tyrosine-phosphorylated receptors (52), have cytoplasmic tails that are thought to be intrinsically disordered with a continuum of states that can exhibit high molecular entropy. The binding of a modifying enzyme to a disordered substrate incurs an entropic penalty because binding is often compatible with only a subset of all states. If phosphorylation induces local order, it may increase the effective on-rate for substrate binding. We note that a switchlike response is not introduced by this mechanism if it applies equally to both modifying enzymes as we have implemented. But a switchlike response can be generated if, for example, the enhancement applies selectively to one modifying enzyme or if the reduced entropic binding allows the substrate to be sequestered away from both modifying enzymes (6,7,13).

Another mechanism that may explain phosphorylation priming across ITAMs is the electrostatically mediated association of the cytoplasmic tails of transmembrane receptors with the membrane. Such interactions have been demonstrated for the CD3*E* subunit of the TCR complex (53,54), the TCR ζ-chain (25), and the ITIM-containing receptor PECAM-1 (55). Because multisite phosphorylation drastically alters the charge of molecules it may induce dissociation of the cytoplasmic tails from the membrane. This mechanism can induce phosphorylation priming if the cytoplasmic tail becomes more accessible to the modifying enzymes when dissociated from the membrane. We note that published models of multisite phosphorylation have often reported switchlike responses, but not changes in potency (8–15). Our previous work has shown that local rebinding can lead to switchlike response when diffusion is limited, but we did not observe a change in potency (12).

Previous experimental work has reported switchlike responses by multisite phosphorylation that can apply to unstructured proteins such as the TCR ζ-chain. For example, phosphorylation has been shown to generate a switchlike response in the membrane localization of the scaffold Ste5 by bulk electrostatics, whereby phosphorylation increases the negative charge of the substrate and therefore leads to reduced membrane association (56,57). As discussed above, the association of the TCR ζ-chain with the membrane is thought to be regulated by bulk electrostatics (25,53). A key difference between Ste5 and the TCR is that bulk electrostatics can completely alter the localization of Ste5 whereas bulk electrostatics may only modestly alter the average distance of the TCR cytoplasmic tails from the membrane.

Although frequently responsible for switchlike responses in other proteins, we have found that multisite phosphorylation of the TCR ζ-chain does not modulate the switchlike response but can modulate the potency and maximum phosphorylation of the receptor. A key difference between the TCR and other multisite substrates known to exhibit switchlike responses is that phosphorylation sites in the latter case are thought to act in concert. For example, in the case of Ste5, Sic1, and NFAT, multiple phosphorylation sites collectively regulate association with the membrane (56,57), association with another protein (58), or a conformational change (59,60), respectively. In contrast, the 10 ITAMs on the TCR can each independently provide docking sites for the effector kinase ZAP-70, which can propagate downstream signaling (20). In this way the TCR, and possibly other immune receptors, have acquired multiple phosphorylation sites that may individually act to amplify ligand binding events.

SUPPORTING MATERIAL

Supporting Materials and Methods, eight figures, and three tables are available at http://www.biophysj.org/biophysj/supplemental/S0006-3495(16)30104-7.

AUTHOR CONTRIBUTIONS

H.M., B.d.W., P.A.v.d.M., and O.D. designed research; H.M., B.d.W., L.C., J.A., and O.D. performed research; H.M., B.d.W., L.C., J.A., P.K.M., P.A.v.d.M., and O.D. analyzed data; and H.M., B.d.W., P.A.v.d.M., and O.D. wrote the article.

ACKNOWLEDGMENTS

We acknowledge Shaun-Paul Cordoba, Jesse Goyette, Laura Makin, and Adrian Yemm for assistance in developing the experimental protocol. We thank Vahid Shahrezaei and David Vaux for feedback during the development of the work.

O.D. is funded by a Sir Henry Dale Fellowship jointly funded by the Wellcome Trust and the Royal Society (grant No. 098363), P.A.v.d.M. is funded by a Wellcome Trust Senior Investigator Award (grant No. 101799), and J.A. is funded by an National Science Foundation Career Grant (grant No. DMS-1454739). We acknowledge support from the Medical Research Council (grant No. MR/J002011/1).

SUPPORTING CITATIONS

References (61–65) appear in the Supporting Material.

REFERENCES

1. Cohen, P. 2001. The role of protein phosphorylation in human health and disease: The Sir Hans Krebs Medal Lecture. *Eur. J. Biochem.* 268:5001–5010.
2. Prabakaran, S., G. Lippens, …, J. Gunawardena. 2012. Post-translational modification: nature's escape from genetic imprisonment and the basis for dynamic information encoding. *Wiley Interdiscip. Rev. Syst. Biol. Med.* 4:565–583.
3. Östman, A., C. Hellberg, and F. D. Böhmer. 2006. Protein-tyrosine phosphatases and cancer. *Nat. Rev. Cancer.* 6:307–320.
4. Gnad, F., S. Ren, …, M. Mann. 2007. PHOSIDA (phosphorylation site database): management, structural and evolutionary investigation, and prediction of phosphosites. *Genome Biol.* 8:R250.
5. Hlavacek, W. S., J. R. Faeder, …, W. Fontana. 2006. Rules for modeling signal-transduction systems. *Sci. STKE.* 2006:re6.
6. Salazar, C., and T. Höfer. 2009. Multisite protein phosphorylation—from molecular mechanisms to kinetic models. *FEBS J.* 276:3177–3198.
7. Ferrell, J. E., Jr., and S. H. Ha. 2014. Ultrasensitivity part II: multisite phosphorylation, stoichiometric inhibitors, and positive feedback. *Trends Biochem. Sci.* 39:556–569.
8. Liu, X., L. Bardwell, and Q. Nie. 2010. A combination of multisite phosphorylation and substrate sequestration produces switchlike responses. *Biophys. J.* 98:1396–1407.
9. Wang, L., Q. Nie, and G. Enciso. 2010. Nonessential sites improve phosphorylation switch. *Biophys. J.* 99:L41–L43.
10. Malleshaiah, M. K., V. Shahrezaei, …, S. W. Michnick. 2010. The scaffold protein Ste5 directly controls a switch-like mating decision in yeast. *Nature.* 465:101–105.
11. Martins, B. M. C., and P. S. Swain. 2013. Ultrasensitivity in phosphorylation-dephosphorylation cycles with little substrate. *PLOS Comput. Biol.* 9:e1003175.
12. Dushek, O., P. A. van der Merwe, and V. Shahrezaei. 2011. Ultrasensitivity in multisite phosphorylation of membrane-anchored proteins. *Biophys. J.* 100:1189–1197.
13. Lenz, P., and P. S. Swain. 2006. An entropic mechanism to generate highly cooperative and specific binding from protein phosphorylations. *Curr. Biol.* 16:2150–2155.
14. Mukhopadhyay, H., S.-P. Cordoba, …, O. Dushek. 2013. Systems model of T cell receptor proximal signaling reveals emergent ultrasensitivity. *PLOS Comput. Biol.* 9:e1003004.
15. Salazar, C., and T. Höfer. 2007. Versatile regulation of multisite protein phosphorylation by the order of phosphate processing and protein-protein interactions. *FEBS J.* 274:1046–1061.
16. van der Merwe, P. A., and O. Dushek. 2011. Mechanisms for T cell receptor triggering. *Nat. Rev. Immunol.* 11:47–55.
17. Smith-Garvin, J. E., G. A. Koretzky, and M. S. Jordan. 2009. T cell activation. *Annu. Rev. Immunol.* 27:591–619.
18. Lever, M., P. K. Maini, …, O. Dushek. 2014. Phenotypic models of T cell activation. *Nat. Rev. Immunol.* 14:619–629.
19. Restifo, N. P., M. E. Dudley, and S. A. Rosenberg. 2012. Adoptive immunotherapy for cancer: harnessing the T cell response. *Nat. Rev. Immunol.* 12:269–281.
20. Love, P. E., and S. M. Hayes. 2010. ITAM-mediated signaling by the T-cell antigen receptor. *Cold Spring Harb. Perspect. Biol.* 2:a002485.
21. Secrist, J. P., L. A. Burns, …, R. T. Abraham. 1993. Stimulatory effects of the protein tyrosine phosphatase inhibitor, pervanadate, on T-cell activation events. *J. Biol. Chem.* 268:5886–5893.
22. Huang, J., M. Brameshuber, …, M. M. Davis. 2013. A single peptide-major histocompatibility complex ligand triggers digital cytokine secretion in CD^{4+} T cells. *Immunity.* 39:846–857.
23. Kersh, E. N., A. S. Shaw, and P. M. Allen. 1998. Fidelity of T cell activation through multistep T cell receptor ζ-phosphorylation. *Science.* 281:572–575.
24. van Oers, N. S., B. Tohlen, …, C. A. Slaughter. 2000. The 21- and 23-kD forms of TCR-ζ are generated by specific ITAM phosphorylations. *Nat. Immunol.* 1:322–328.
25. Zhang, H., S.-P. Cordoba, …, P. A. van der Merwe. 2011. Basic residues in the T-cell receptor ζ cytoplasmic domain mediate membrane association and modulate signaling. *Proc. Natl. Acad. Sci. USA.* 108:19323–19328.
26. Choudhuri, K., D. Wiseman, …, P. A. van der Merwe. 2005. T-cell receptor triggering is critically dependent on the dimensions of its peptide-MHC ligand. *Nature.* 436:578–582.
27. Nika, K., C. Soldani, …, O. Acuto. 2010. Constitutively active Lck kinase in T cells drives antigen receptor signal transduction. *Immunity.* 32:766–777.
28. Cordoba, S. P., K. Choudhuri, …, P. A. van der Merwe. 2013. The large ectodomains of CD45 and CD148 regulate their segregation from and inhibition of ligated T-cell receptor. *Blood.* 121:4295–4302.
29. Geiger, T., A. Wehner, …, M. Mann. 2012. Comparative proteomic analysis of eleven common cell lines reveals ubiquitous but varying expression of most proteins. *Mol. Cell. Proteomics.* 11, M111.014050.
30. James, J. R., and R. D. Vale. 2012. Biophysical mechanism of T-cell receptor triggering in a reconstituted system. *Nature.* 487:64–69.
31. Isakov, N., R. L. Wange, …, L. E. Samelson. 1995. ZAP-70 binding specificity to T cell receptor tyrosine-based activation motifs: the tandem SH2 domains of ZAP-70 bind distinct tyrosine-based activation motifs with varying affinity. *J. Exp. Med.* 181:375–380.
32. Hui, E., and R. D. Vale. 2014. In vitro membrane reconstitution of the T-cell receptor proximal signaling network. *Nat. Struct. Mol. Biol.* 21:133–142.
33. Flock, T., R. J. Weatheritt, …, M. M. Babu. 2014. Controlling entropy to tune the functions of intrinsically disordered regions. *Curr. Opin. Struct. Biol.* 26:62–72.
34. Dosztányi, Z., V. Csizmók, …, I. Simon. 2005. The pairwise energy content estimated from amino acid composition discriminates

between folded and intrinsically unstructured proteins. *J. Mol. Biol.* 347:827–839.

35. Xue, B., R. L. Dunbrack, …, V. N. Uversky. 2010. PONDR-FIT: a meta-predictor of intrinsically disordered amino acids. *Biochim. Biophys. Acta.* 1804:996–1010.

36. Boal, D. 2012. Mechanics of the Cell. Cambridge University Press, New York.

37. Rawat, N., and P. Biswas. 2009. Size, shape, and flexibility of proteins and DNA. *J. Chem. Phys.* 131:165104.

38. Irles, C., A. Symons, …, O. Acuto. 2003. CD45 ectodomain controls interaction with GEMs and Lck activity for optimal TCR signaling. *Nat. Immunol.* 4:189–197.

39. Ottinger, E. A., M. C. Botfield, and S. E. Shoelson. 1998. Tandem SH2 domains confer high specificity in tyrosine kinase signaling. *J. Biol. Chem.* 273:729–735.

40. Klammt, C., L. Novotná, …, B. F. Lillemeier. 2015. T cell receptor dwell times control the kinase activity of Zap70. *Nat. Immunol.* 16:961–969.

41. McKeithan, T. W. 1995. Kinetic proofreading in T-cell receptor signal transduction. *Proc. Natl. Acad. Sci. USA.* 92:5042–5046.

42. Altan-Bonnet, G., and R. N. Germain. 2005. Modeling T cell antigen discrimination based on feedback control of digital ERK responses. *PLoS Biol.* 3:e356.

43. Dushek, O., R. Das, and D. Coombs. 2009. A role for rebinding in rapid and reliable T cell responses to antigen. *PLOS Comput. Biol.* 5:e1000578.

44. Dushek, O., M. Aleksic, …, P. A. van der Merwe. 2011. Antigen potency and maximal efficacy reveal a mechanism of efficient T cell activation. *Sci. Signal.* 4:ra39.

45. Dushek, O., and P. A. van der Merwe. 2014. An induced rebinding model of antigen discrimination. *Trends Immunol.* 35:153–158.

46. Zikherman, J., and B. Au-Yeung. 2015. The role of T cell receptor signaling thresholds in guiding T cell fate decisions. *Curr. Opin. Immunol.* 33:43–48.

47. Holst, J., H. Wang, …, D. A. Vignali. 2008. Scalable signaling mediated by T cell antigen receptor-CD3 ITAMs ensures effective negative selection and prevents autoimmunity. *Nat. Immunol.* 9:658–666.

48. Hwang, S., A. C. Palin, …, P. E. Love. 2015. TCR ITAM multiplicity is required for the generation of follicular helper T-cells. *Nat. Commun.* 6:6982.

49. Coba, M. P., A. J. Pocklington, …, S. G. N. Grant. 2009. Neurotransmitters drive combinatorial multistate postsynaptic density networks. *Sci. Signal.* 2:ra19.

50. Travers, T., H. Shao, …, C. J. Camacho. 2015. Tandem phosphorylation within an intrinsically disordered region regulates ACTN4 function. *Sci. Signal.* 8:ra51.

51. Sjölin-Goodfellow, H., M. P. Frushicheva, …, A. Weiss. 2015. The catalytic activity of the kinase ZAP-70 mediates basal signaling and negative feedback of the T cell receptor pathway. *Sci. Signal.* 8:ra49.

52. Dushek, O., J. Goyette, and P. A. van der Merwe. 2012. Non-catalytic tyrosine-phosphorylated receptors. *Immunol. Rev.* 250:258–276.

53. Xu, C., E. Gagnon, …, K. W. Wucherpfennig. 2008. Regulation of T cell receptor activation by dynamic membrane binding of the CD3epsilon cytoplasmic tyrosine-based motif. *Cell.* 135:702–713.

54. Shi, X., Y. Bi, …, C. Xu. 2013. Ca^{2+} regulates T-cell receptor activation by modulating the charge property of lipids. *Nature.* 493:111–115.

55. Paddock, C., B. L. Lytle, …, D. K. Newman. 2011. Residues within a lipid-associated segment of the PECAM-1 cytoplasmic domain are susceptible to inducible, sequential phosphorylation. *Blood.* 117:6012–6023.

56. Strickfaden, S. C., M. J. Winters, …, P. M. Pryciak. 2007. A mechanism for cell-cycle regulation of MAP kinase signaling in a yeast differentiation pathway. *Cell.* 128:519–531.

57. Serber, Z., and J. E. Ferrell, Jr. 2007. Tuning bulk electrostatics to regulate protein function. *Cell.* 128:441–444.

58. Nash, P., X. Tang, …, M. Tyers. 2001. Multisite phosphorylation of a CDK inhibitor sets a threshold for the onset of DNA replication. *Nature.* 414:514–521.

59. Okamura, H., J. Aramburu, …, A. Rao. 2000. Concerted dephosphorylation of the transcription factor NFAT1 induces a conformational switch that regulates transcriptional activity. *Mol. Cell.* 6:539–550.

60. Salazar, C., and T. Höfer. 2003. Allosteric regulation of the transcription factor NFAT1 by multiple phosphorylation sites: a mathematical analysis. *J. Mol. Biol.* 327:31–45.

61. Reeves, D., K. Cheveralls, and J. Kondev. 2011. Regulation of biochemical reaction rates by flexible tethers. *Phys. Rev. E Stat. Nonlin. Soft Matter Phys.* 84:021914.

62. Kutys, M. L., J. Fricks, and W. O. Hancock. 2010. Monte Carlo analysis of neck linker extension in kinesin molecular motors. *PLOS Comput. Biol.* 6:e1000980.

63. van Valen, D., M. Haataja, and R. Phillips. 2009. Biochemistry on a leash: the roles of tether length and geometry in signal integration proteins. *Biophys. J.* 96:1275–1292.

64. Andrews, S. S. 2014. Methods for modeling cytoskeletal and DNA filaments. *Phys. Biol.* 11:011001.

65. Milner, S., T. Witten, and M. Cates. 1988. Theory of the grafted polymer brush. *Macromolecules.* 21:2610–2619.

Biophysical Journal, Volume *110*

Supplemental Information

Multisite Phosphorylation Modulates the T Cell Receptor ζ-Chain Potency but not the Switchlike Response

Himadri Mukhopadhyay, Ben de Wet, Lara Clemens, Philip K. Maini, Jun Allard, P. Anton van der Merwe, and Omer Dushek

Supporting Material:
Multisite Phosphorylation Modulates T cell Receptor ζ-chain Potency but not the Switch-like Response

Himadri Mukhopadhyay[†*], Ben de Wet[†*], Lara Clemens[^], Philip K. Maini[‡], Jun Allard[^], P. Anton van der Merwe[†,¶], Omer Dushek[†,‡,¶]

[†]Sir William Dunn School of Pathology, University of Oxford, Oxford, Oxfordshire, United Kingdom
[‡]Wolfson Centre for Mathematical Biology, Mathematical Institute, University of Oxford, Oxford, Oxfordshire, United Kingdom
[^]Department of Mathematics, University of California, Irvine, United States
* These authors contributed equally to this work
[¶]Corresponding authors

Supporting Text

Mathematical model of multisite phosphorylation

The mathematical model includes a multisite substrate (S, TCR ζ-chain) that is phosphorylated by a kinase (E, Lck) and dephosphorylated by a phosphatase (F, CD148) by an unstructured random mechanism. We coarse-grained the tyrosines in each of the 6 ITAMs present in the ζ-chain dimer into a single effective site. Each elementary modification reaction is governed by an on-rate (k_{on}), an off-rate (k_{off}), and by a catalytic rate (k_{cat}),

$$\begin{aligned} E + S_j &\underset{k_{\text{off}}}{\overset{(N-j)k_{\text{on}}\lambda^j}{\rightleftharpoons}} ES_j \overset{k_{\text{cat}}}{\rightarrow} E + S_{j+1} \\ F + S_j &\underset{k_{\text{off}}}{\overset{jk_{\text{on}}\lambda^j}{\rightleftharpoons}} FS_j \overset{k_{\text{cat}}}{\rightarrow} F + S_{j-1} \end{aligned} \quad (1)$$

where N is the number of ITAMs ($N = 6$ in this case) and S_j is the concentration of substrate phosphorylated on j ITAMs. We include a phosphorylation-dependent enhancement in the enzymatic affinity by increasing k_{on} by a factor of λ for each phosphorylated site for both enzymes. The normalised total phosphorylation of the substrate was calculated at steady-state as follows,

$$\text{Phosphorylation} = \sum_{j=0}^{N} j(S_j + ES_j + FS_j)/(6S_T)$$

where S_T is the total concentration of substrate. We assume for all calculations that the reaction rates are identical for both modifying enzymes.

The system of ordinary-differential-equations (ODEs) representing the biochemical reaction network based on this scheme was generated in BioNetGen (1) and integrated in Matlab (Mathworks, MA). The parameters used are $k_{\text{off}} = 1\ \text{s}^{-1}$, $k_{\text{cat}} = 10\ \text{s}^{-1}$, and $S_T = 1\ \mu\text{m}^{-2}$ with the indicated variation to k_{on} ($\mu\text{m}^{-2}\text{s}^{-1}$) and λ (dimensionless). The ratio of E to F was varied by varying F over a 10^6-fold range to obtain the dose-response.

Mathematical model of polymer configurations

Polymer model for the TCR ζ-chain. Disordered chains of amino acids are often modeled as freely-jointed chains as these models agree favourably with experiments (2–4). In these models, each amino acid is assumed to be a rigid rod of length $\delta = 0.3$ nm, referred to as the Kuhn length, connected by a joint that freely explores configurations in three dimensions. The persistence length, an alternative quantity measuring protein flexibility, is equal to half the Kuhn length (5).

The murine ζ-chain has 113 amino acids in its cytoplasmic tails with tyrosines at positions 72/83 (ζ1 ITAM), 111/123 (ζ2 ITAM), and 142/153 (ζ3 ITAM). Assuming a standard disordered chain leads to $N = 113$ segments with individual segment lengths (or Kuhn lengths) of $\delta = 0.3$ nm. To explore the possibility that the chain has some ordered structure, we assume that the contour length $L_c = \delta N \approx 33.9$ nm is fixed but explore a range of Kuhn lengths. This ordered structure could arise because of inter-residue interactions in the native unphosphorylated state or following post-translational modification, such as tyrosine phosphorylation. We note that in these calculations phosphorylation of a site on one ζ-chain in the dimer only induces local order and does not induce ordering of the other chain.

To model interaction of the ζ-chain with its enzymes, we estimate the geometric properties of the catalytic domains of Lck and CD148. The volume of the catalytic domain of Lck is 45 nm^3 based on its crystal structure. Alternatively, the volume can be estimated based on an estimate for protein density, which is approximately 1.41g / cm^3. Using this value and the fact that the catalytic domain consists of 254 amino acids whose molecular mass is 29 kDa, we estimate the volume as follows,

$$(29 \times 1000\text{Da}) * (1.66 \times 10^{-27}\text{kg / Da}) * (1000\text{g / kg})/(1.41\text{g / cm}^3) = 34\text{nm}^3. \tag{2}$$

This estimate is similar to the estimate based on the crystal structure. Approximating the domain as a sphere, we arrive at a radius $r_K \approx 3.2 - 3.5$nm. Given that the catalytic domain of CD148 is similar in size, we assume for simplicity that it has similar dimensions.

Calculation of occlusion probability. We perform Monte Carlo simulation on the polymer model described above using the Metropolis algorithm, which generates an ensemble of polymer configurations at constant temperature (6). At each configuration in the ensemble we determine whether the enzyme is occluded from its binding site, which occurs with probability P. The binding site represents a tyrosine that is to be modified at a specific location along the length of the polymer.

From the thermodynamic detailed balance condition (6), the dissociation constants $K \equiv k_{\text{off}}/k_{\text{on}}$ of the floppy (native) state (K_F) and the rigid (partially phosphorylated) state (K_R) are related by

$$\frac{K_R}{K_F} = \exp\left(\frac{G_R - G_F}{k_B T}\right) \tag{3}$$

$$= \exp\left(\frac{S_F - S_R}{k_B}\right) \tag{4}$$

$$= \frac{P_F}{P_R} \tag{5}$$

where $G_j = E_j - TS_j$ is the free energy of binding in the floppy or rigid state, $S_j = k_B \ln P_j$ is the molecular entropy of binding, and P_j is the probability in the canonical ensemble that the configuration allows for binding.

To estimate the maximum enhancement in binding (λ_{max}) we compare the probability of binding in the floppy state to the probability of binding when the ζ-chain polymer is perfectly rigid (i.e. with $N = 1$ segments whose Kuhn length is $\delta = L_c = 33.9$ nm). In this latter limit the enzyme is never occluded by the rest of the polymer (or the other polymer in the dimer) and therefore $P_R = 1$. It follows that λ_{max} can be calculated based on P_F as follows,

$$\lambda_{\text{max}} = \frac{K_F}{K_R} = 1/P_F. \tag{6}$$

Polymer model variants. The binding enhancement depends on the location of binding site along the polymer. We find that λ_{max} is maximal for binding sites near the midpoint of the polymer $i = N/2$ with a weak dependence on binding sites that are distal to the midpoint (Fig. 5D). We note that binding sites in the simulations are located at segment joints leading to apparent discontinuities (Fig. 5D).

The polymer simulations were performed in free space but since the TCR ζ-chain is a transmembrane protein, we anticipated that an interaction with the membrane would alter the number of available configurations and therefore our estimates of λ_{max}. Therefore, we modified the simulation to include a two-dimensional plane with the TCR ζ-chain polymer embedded therein and stipulated that neither the polymer nor the enzyme are able to cross the plane (membrane). Surprisingly, we find a weak dependence on the presence of the membrane that results from two competing effects (not shown). First, the membrane leads to more configurations in which the enzyme is sterically blocked from its site (increasing λ_{max}). Second, the polymer tends to straighten out when constrained by a plane (7) (decreasing λ_{max}). The net effect is a weak dependence on the presence of a membrane.

BioNetGen Script

```
# A substrate with six ITAMs sites is modified by a kinase (E) and a phosphatase (F).
# Modifications follow a random phosphorylation scheme.

begin parameters

# Parameter 'place holders' are defined in BioNetGen, and these are modified in MATLAB
# to include, for example, phosphorylation-dependent on-rates (Fig . 4A)

# kinase on rates for each ITAM

ek_on1 1
ek_on2 1
ek_on3 1
ek_on4 1
ek_on5 1
ek_on6 1

# phosphatase on rates for each ITAM

fk_on1 1
fk_on2 1
fk_on3 1
fk_on4 1
fk_on5 1
fk_on6 1
```

```
# kinase off rates for each ITAM

ek_off1 1
ek_off2 1
ek_off3 1
ek_off4 1
ek_off5 1
ek_off6 1

# phosphatase off rates for each ITAM

fk_off1 1
fk_off2 1
fk_off3 1
fk_off4 1
fk_off5 1
fk_off6 1

# kinase rates of catalysis

ek_cat1 1
ek_cat2 1
ek_cat3 1
ek_cat4 1
ek_cat5 1
ek_cat6 1

# phosphatase rates of catalysis

fk_cat1 1
fk_cat2 1
fk_cat3 1
fk_cat4 1
fk_cat5 1
fk_cat6 1

# total amounts of substrate, kinase and phosphatase

S_T 100
E_T 1e3
F_T 1e3

end parameters

# Define molecule types.
# Multisite substrate (S) can be fully dephosphorylated (U), phosphorylated on one ITAM (P),
# phosphorylated on two ITAMs (2P), etc.
# Only catalytic domains are defined for the kinase (ecat) and phosphatase (fcat).

begin molecule types

S(Y~U~P~2P~3P~4P~5P~6P)
E(ecat)
F(fcat)

end molecule types
```

```
# Define initial conditions.

begin seed species

S(Y~U) S_T
E(ecat) E_T
F(fcat) F_T

end seed species

# Define reaction network.

begin reaction rules

# fully dephosphorylated substrate <-> substrate phosphorylated on one ITAM

E(ecat) + S(Y~U) <-> E(ecat!1).S(Y~U!1) ek_on1, ek_off1
E(ecat!1).S(Y~U!1) -> E(ecat) + S(Y~P) ek_cat1

F(fcat) + S(Y~P) <-> F(fcat!1).S(Y~P!1) fk_on1, fk_off1
F(fcat!1).S(Y~P!1) -> F(fcat) + S(Y~U) fk_cat1

# substrate phosphorylated on one ITAM <-> substrate phosphorylated on two ITAMs

E(ecat) + S(Y~P) <-> E(ecat!1).S(Y~P!1) ek_on2, ek_off2
E(ecat!1).S(Y~P!1) -> E(ecat) + S(Y~2P) ek_cat2

F(fcat) + S(Y~2P) <-> F(fcat!1).S(Y~2P!1) fk_on2, fk_off2
F(fcat!1).S(Y~2P!1) -> F(fcat) + S(Y~P) fk_cat2

# substrate phosphorylated on two ITAMs <-> substrate phosphorylated on three ITAMs

E(ecat) + S(Y~2P) <-> E(ecat!1).S(Y~2P!1) ek_on3, ek_off3
E(ecat!1).S(Y~2P!1) -> E(ecat) + S(Y~3P) ek_cat3

F(fcat) + S(Y~3P) <-> F(fcat!1).S(Y~3P!1) fk_on3, fk_off3
F(fcat!1).S(Y~3P!1) -> F(fcat) + S(Y~2P) fk_cat3

# substrate phosphorylated on three ITAMs <-> substrate phosphorylated on four ITAMs

E(ecat) + S(Y~3P) <-> E(ecat!1).S(Y~3P!1) ek_on4, ek_off4
E(ecat!1).S(Y~3P!1) -> E(ecat) + S(Y~4P) ek_cat4

F(fcat) + S(Y~4P) <-> F(fcat!1).S(Y~4P!1) fk_on4, fk_off4
F(fcat!1).S(Y~4P!1) -> F(fcat) + S(Y~3P) fk_cat4

# substrate phosphorylated on four ITAMs <-> substrate phosphorylated on five ITAMs

E(ecat) + S(Y~4P) <-> E(ecat!1).S(Y~4P!1) ek_on5, ek_off5
E(ecat!1).S(Y~4P!1) -> E(ecat) + S(Y~5P) ek_cat5

F(fcat) + S(Y~5P) <-> F(fcat!1).S(Y~5P!1) fk_on5, fk_off5
F(fcat!1).S(Y~5P!1) -> F(fcat) + S(Y~4P) fk_cat5

# substrate phosphorylated on five ITAMs <-> substrate phosphorylated on six ITAMs
```

```
E(ecat) + S(Y~5P) <-> E(ecat!1).S(Y~5P!1) ek_on6, ek_off6
E(ecat!1).S(Y~5P!1) -> E(ecat) + S(Y~6P) ek_cat6

F(fcat) + S(Y~6P) <-> F(fcat!1).S(Y~6P!1) fk_on6, fk_off6
F(fcat!1).S(Y~6P!1) -> F(fcat) + S(Y~5P) fk_cat6


end reaction rules

# Define observables.

begin observables

1 Molecules Szero S(Y~U!?)
2 Molecules Sone S(Y~P!?)
3 Molecules Stwo S(Y~2P!?)
4 Molecules Sthree S(Y~3P!?)
5 Molecules Sfour S(Y~4P!?)
6 Molecules Sfive S(Y~5P!?)
7 Molecules Ssix S(Y~6P!?)

end observables


generate_network({overwrite=>1});

writeMfile({});
```

Supporting References

1. Hlavacek, W. S., J. R. Faeder, M. L. Blinov, R. G. Posner, M. Hucka, and W. Fontana, 2006. Rules for modeling signal-transduction systems. *Science's STKE : signal transduction knowledge environment* 2006:re6.

2. Reeves, D., K. Cheveralls, and J. Kondev, 2011. Regulation of biochemical reaction rates by flexible tethers. *Physical Review E - Statistical, Nonlinear, and Soft Matter Physics* 84:1–12.

3. Kutys, M. L., J. Fricks, and W. O. Hancock, 2010. Monte Carlo analysis of neck linker extension in kinesin molecular motors. *PLoS computational biology* 6:e1000980.

4. Van Valen, D., M. Haataja, and R. Phillips, 2009. Biochemistry on a leash: the roles of tether length and geometry in signal integration proteins. *Biophysical journal* 96:1275–92.

5. Andrews, S. S., 2014. Methods for modeling cytoskeletal and DNA filaments. *Physical Biology* 11:011001.

6. Boal, D., 2012. Mechanics of the Cell. Cambridge University Press.

7. Milner, S., T. Witten, and M. Cates, 1988. Theory of the grafted polymer brush. *Macromolecules* 21:2610–2619.

Supporting Tables

	Hill Number (normalised)			$log_{10}(EC_{50})$ (normalised)		
	Mean	SEM	N	Mean	SEM	N
ζ123	1.000	0.225	5	0.000	0.040	9
ζX23	1.221	0.244	5	0.121	0.033	9
ζ1X3	1.505	0.359	5	0.137	0.036	9
ζ12X	1.454	0.354	5	0.074	0.047	9
ζ1XX	1.237	0.228	5	0.184	0.042	9
ζX2X	1.243	0.249	5	0.191	0.028	8
ζXX3	1.065	0.174	5	0.188	0.034	8

Table S1: Hill Numbers and $\log_{10}(EC_{50})$ for the data in Fig. 2

	Hill Number (normalised)			$log_{10}(EC_{50})$ (normalised)		
	Mean	SEM	N	Mean	SEM	N
ζ123	1.000	0.152	5	0.000	0.013	5
ζX23	1.519	0.164	4	0.093	0.038	4
ζ1X3	1.361	0.258	3	0.072	0.048	3
ζ12X	0.915	0.160	3	0.041	0.023	3
ζ1XX	0.927	0.158	3	0.155	0.025	3
ζX2X	0.978	0.154	3	0.183	0.022	3
ζXX3	0.821	0.127	4	0.199	0.013	4

Table S2: Hill Numbers and $\log_{10}(EC_{50})$ for the data in Fig. 3

	Hill Number (normalised)			$log_{10}(EC_{50})$ (normalised)		
	Mean	SEM	N	Mean	SEM	N
ζ123	1.000	0.173	4	0.000	0.023	4
ζX23	1.066	0.153	4	0.048	0.015	4
ζ1X3	1.062	0.184	4	0.032	0.034	4
ζ12X	1.097	0.238	4	0.041	0.029	4
ζ1XX	1.036	0.117	4	0.105	0.012	4
ζX2X	1.070	0.105	4	0.113	0.047	4
ζXX3	0.912	0.212	4	0.163	0.047	4

Table S3: Hill Numbers and $\log_{10}(EC_{50})$ for the data in Fig. S5

Supporting Figures

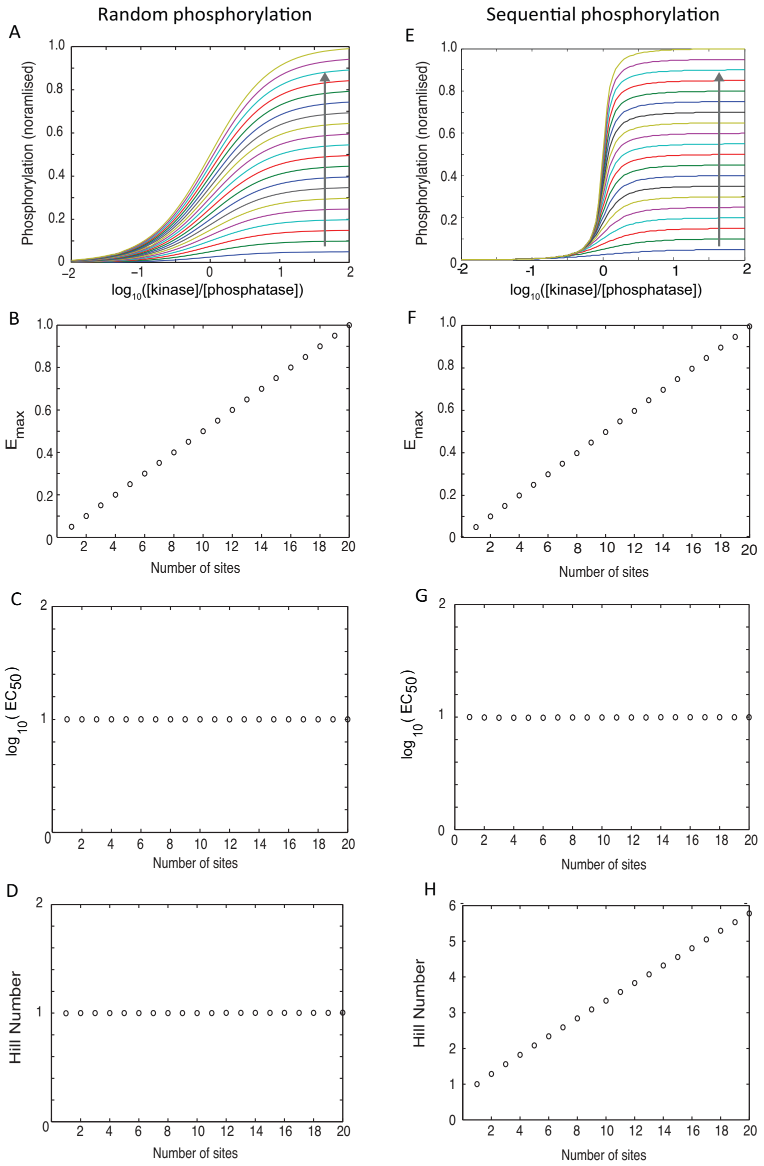

Fig S1: **Phosphorylation profiles of multisite phosphorylation based on a random and sequential phosphorylation mechanism.** Mathematical model of a substrate containing 1, 2, 3, ... 20 phosphorylation sites (arrow indicates direction of increasing number of sites) that can be phosphorylated by a kinase and dephosphorylated by a phosphatase in any order (A-D, Random phosphorylation) or in a strict sequential order (E-H, Sequential phosphorylation). Phosphorylation (y-axis in A, E) is the total phosphorylation calculated by adding the concentration of substrate phosphorylated on N sites multiplied by N normalised to the maximum phosphorylation of a 20-site substrate (see Fig. 4A). Multisite phosphorylation does not modulate potency (EC_{50}) when phosphorylation is random or sequential but does modulate the switch-like response when phosphorylation is sequential.

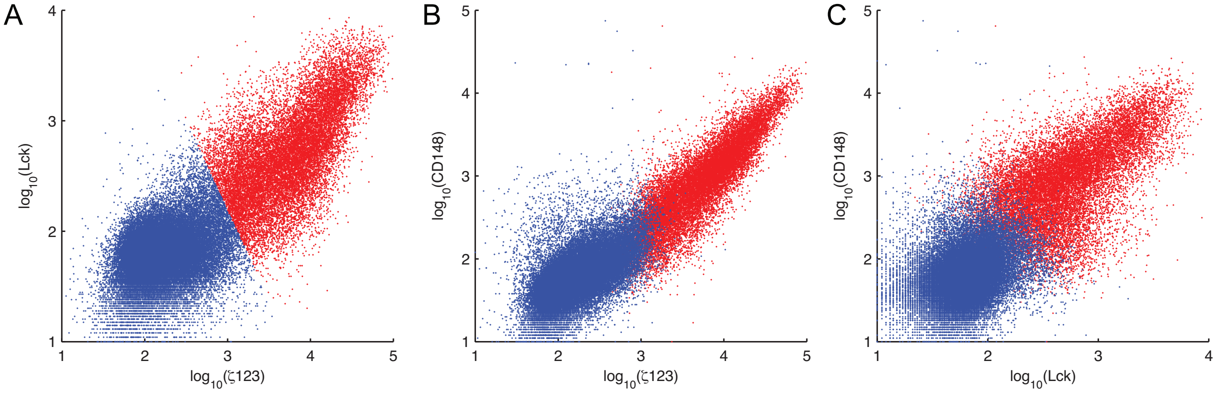

Fig S2: **Two-dimensional projections of expression data shown in Fig. 1B.** A-C) Pairwise projections of the three-dimensional molecular expression for ζ123, Lck, and CD148.

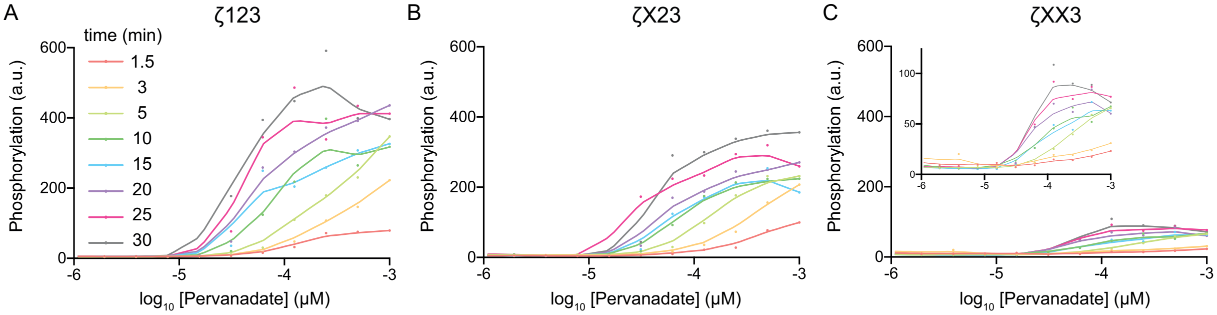

Fig S3: **Phosphorylation timecourse for modules with different number of phosphorylation sites follow similar kinetics to steady-state.** Phosphorylation for reconstitution of Lck, CD148 and A) ζ123, B) ζX23, and C) ζXX3 at the indicated time. Solid line is a running mean of the experimental data (filled circles).

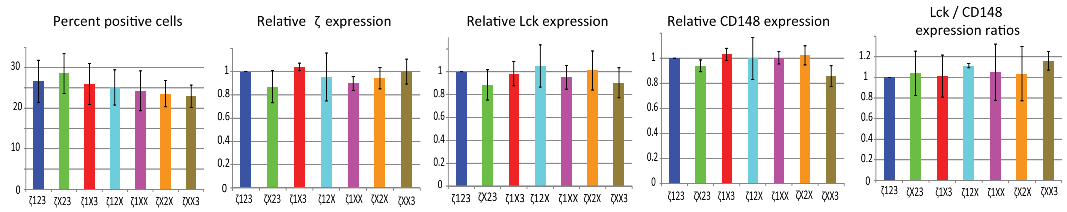

Fig S4: **Mean percent positive and mean molecular expression levels for indicated components for each reconstituted signalling module shown in Fig. 2B,C.** Expression is calculated only for the positive cells as determined by a clustering algorithm (see Fig. 1B) and normalised to the wild-type ζ-chain in each case (ζ123).

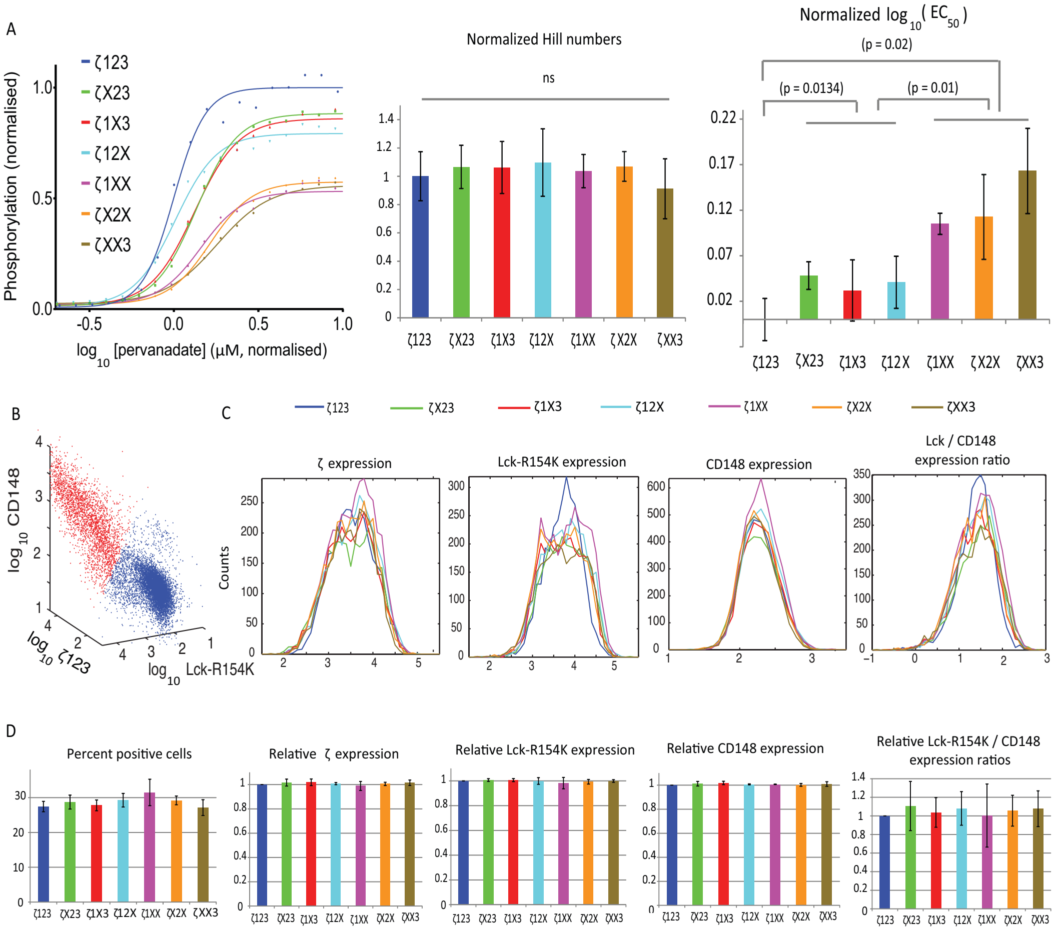

Fig S5: **Modulation of potency and not the switch-like response by multisite phosphorylation is independent of the Lck SH2 domain.** A) Phosphorylation profiles of reconstituted signalling modules containing Lck with a point mutation in the SH2 domain that abolishes binding (Lck-R154K), CD148, and either wild-type ζ-chain (ζ123) or all possible ITAM mutations as indicated. A Hill function is fit to all curves to produce estimates of the potency (EC_{50}) and the Hill number. B-D) Analysis of the expression of each component reveals comparable expression levels for each of the 7 reconstituted signalling modules. Representative clustering of cells as positive (red) or negative (blue) for expression of all 3 components along with C) representative expression profiles of each component in all 7 reconstituted signalling modules. D) Mean percent positive and mean expression of the indicated component (from the positive population) relative to the signalling module with wild-type ζ-chain (ζ123, blue). Representative data and averaged parameters are normalised to the index module (Lck-R154K, CD148, and ζ123) with error bars indicating $\pm$sem. See Materials & Methods for details on normalisation and statistical analysis.

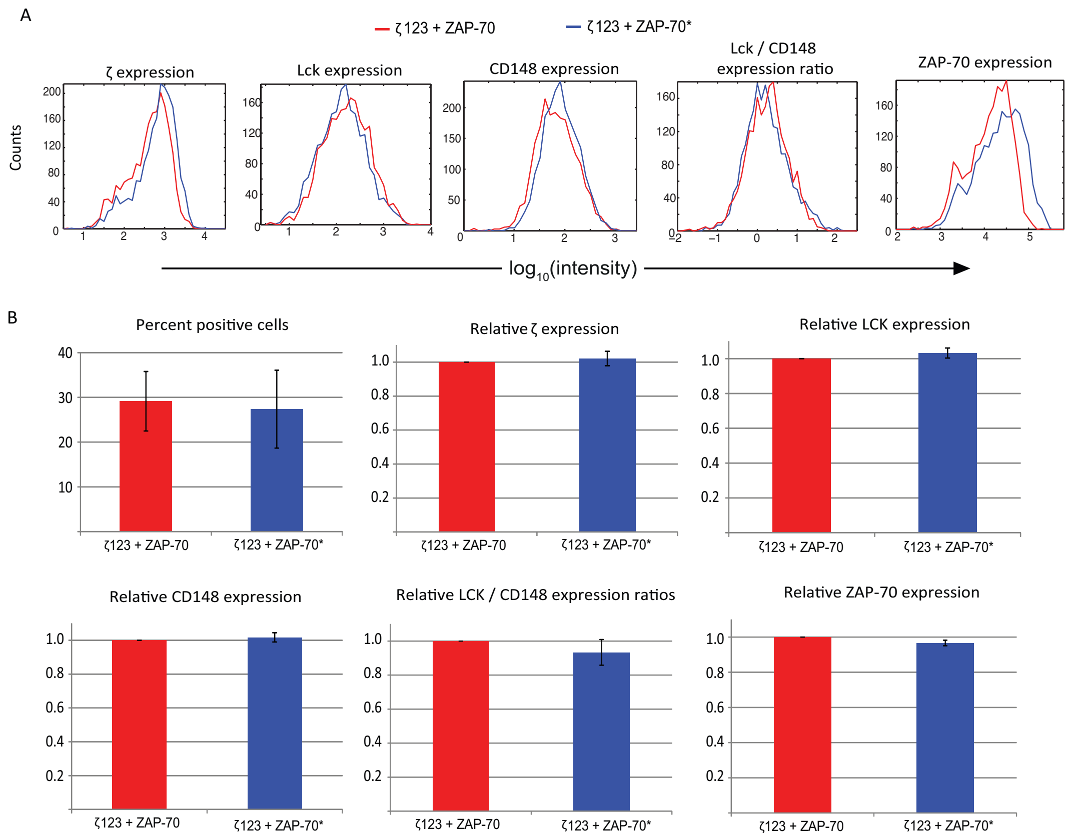

Fig S6: **Molecular expression levels for reconstituted signalling modules shown in Fig 3A-C.** A) Representative flow cytometery data showing expression of the indicated component and the ratio of Lck to CD148 in individual cells. B) Mean percent positive and mean expression of the indicated component (from the positive population) relative to the signalling module with wild-type ZAP-70 (red). The data are taken from at least 3 independent experiments and error bars indicate ±sem.

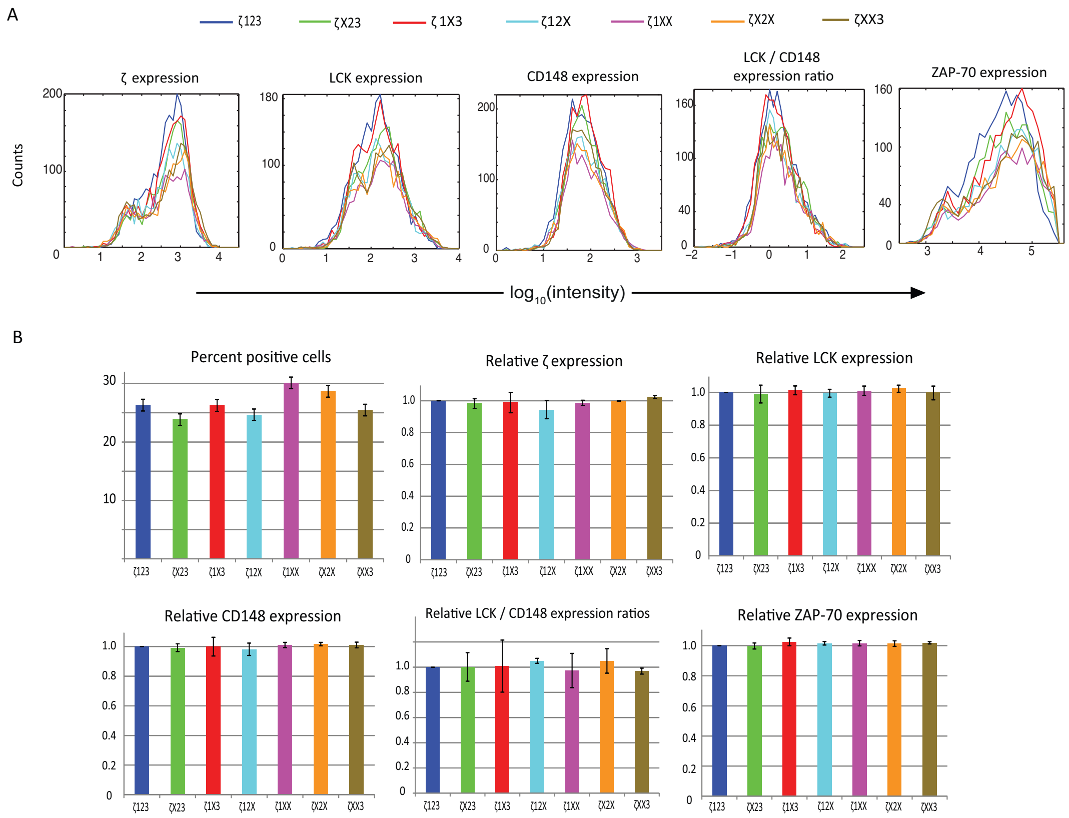

Fig S7: **Molecular expression levels for reconstituted signalling modules shown in Fig. 3D-F.** A) Representative flow cytometry data showing expression of the indicated component and the ratio of Lck to CD148 in individual cells. B) Mean percent positive and mean expression of the indicated component (from the positive population) relative to the signalling module with wild-type ζ-chain (ζ123, blue). The data are taken from at least 3 independent experiments and error bars indicate $\pm$sem.

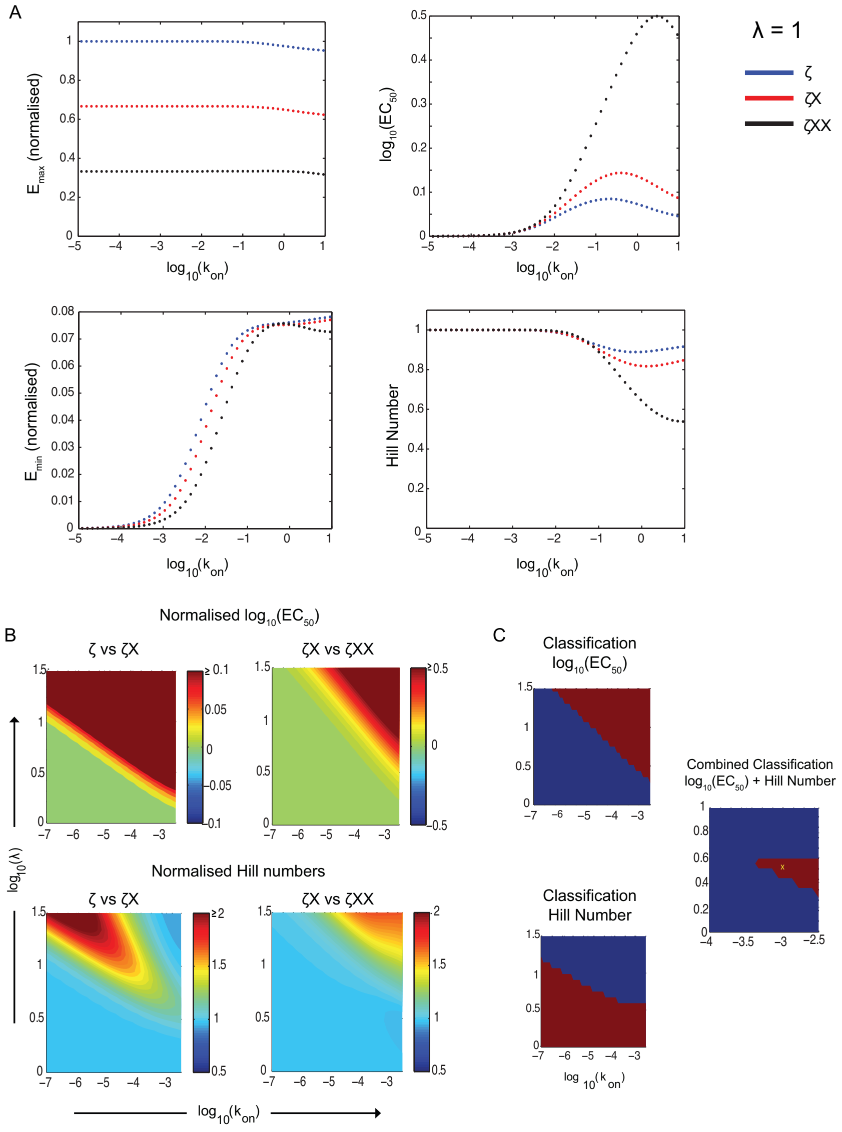

Fig S8: **Parameter scan for multisite phosphorylation model in Fig. 4A,B**. A) In the standard model without a phosphorylation-dependent enhancement ($\lambda = 1$) a change in potency can be observed when the enzymatic affinity is large ($k_{\text{on}} > 0.01\ \mu\text{m}^{-2}\text{s}^{-1}$) but in this case the large affinity of the enzymes to the substrate results in increased basal phosphorylation (E_{min}) which is not observed in the experimental data. B) Heat maps showing the difference in potency (top, on a log-scale) and the fold change in the Hill number (bottom) for different values of the enhancement factor (λ) and the affinities of the enzymes (modified by increasing k_{on}). Comparisons are shown between the 6 site (ζ) and 4 site (ζX) substrate and between the 4 site (ζX) and 2 site (ζXX) substrate. C) Binary maps of the region of $\lambda - k_{\text{on}}$ parameter space where the experimental trends are satisfied for the potency and Hill number (shown in red). These binary classifications are multiplied to obtain the overall combined region of parameter space where both the potency and Hill number trends are satisfied (X denoted the parameter values used to generate the phosphorylation profiles in Fig 4B). See Materials & Methods for computational details.
